# Serum Metabolomic Profiling in a Neonatal Piglet Model of Perinatal Asphyxia: A Pilot Study in Search of Candidate Biomarkers of Acute Hypoxic Injury and Early Post-Resuscitation Recovery

**DOI:** 10.3390/metabo16080554

**Published:** 2026-08-05

**Authors:** Efstathia-Danai Bikouli, Paris Christodoulou, Rozeta Sokou, Eleftheria Karampela, Vasiliki Mougiou, Antigoni Cheilari, Konstantinos Tsiantas, Nikolaos S. Thomaidis, Nicoletta M. Iacovidou, Theodoros Xanthos, Panagiotis Zoumpoulakis

**Affiliations:** 1 Neonatal Intensive Care Unit, General and Maternity Hospital “Helena Venizelou”, 11521 Athens, Greece; 2 Department of Neonatology, Aretaieio Hospital, Medical School, National and Kapodistrian University of Athens, 11528 Athens, Greece; vasiamoug@med.uoa.gr (V.M.); niakobid@med.uoa.gr (N.M.I.); 3 Department of Food Science and Technology, University of West Attica, Ag. Spyridonos, 12243 Egaleo, Greece; pchristodoulou@uniwa.gr (P.C.); ktsiantas@uniwa.gr (K.T.); pzoump@uniwa.gr (P.Z.); 4 Experimental, Educational and Research Center, ELPEN Pharmaceutical, 19009 Pikermi, Greece; ekarampela@elpen.gr; 5 Department of Pharmacognosy and Natural Products Chemistry, Faculty of Pharmacy, National and Kapodistrian University of Athens, Panepistimiopolis Zografou, 17551 Athens, Greece; cheilarianti@pharm.uoa.gr; 6 Laboratory of Analytical Chemistry, Department of Chemistry, National and Kapodistrian University of Athens, Panepistimiopolis Zografou, 15771 Athens, Greece; ntho@chem.uoa.gr; 7 School of Health Sciences, University of West Attica, 12243 Athens, Greece; txanthos@uniwa.gr

**Keywords:** perinatal asphyxia, metabolomics, biomarkers, neonates, resuscitation, ROSC, energy metabolism

## Abstract

**Background/Objectives**: Perinatal asphyxia (PA) is a major cause of neonatal mortality and morbidity both in the short and in the long term. The identification of novel reliable biomarkers is essential in order to improve early diagnosis and allow for accurate prognostication of short- and long-term outcomes. The aim of the current study was to identify serum metabolites substantially affected by PA and resuscitation, using an experimental model in neonate piglets. **Methods**: A prospective, randomized experimental pilot animal study was conducted in 33 neonate Landrace/Large White female piglets, 1–4 days old. Following initial preparation and stabilization, the animals were allocated to three groups. Group A served as the control group while Group B and Group C piglets underwent asphyxia until severe bradycardia or hypotension occurred. Group C animals were subsequently resuscitated, and after return of spontaneous circulation (ROSC), they were stabilized and remained under further monitoring for 30 min. Blood samples for metabolic profiling were obtained at predefined timepoints as defined below. “Baseline” samples were taken from all animals after the initial stabilization; “asphyxia” sampling was performed at the time of hemodynamic compromise, while “final” sampling was performed 1 h after baseline in Group A animals and 30 min post-ROSC in Group C animals. The serum samples obtained were further analyzed using nuclear magnetic resonance (NMR) spectroscopy. **Results**: Distinct metabolic phenotypes were observed between the “baseline” state and asphyxia. Post-resuscitation and post-ROSC, the metabolic phenotype appeared to partially shift back to the “baseline” cluster but remained distinct from both of the other groups. The results were further processed using a structured biomarker discovery pipeline. Key metabolites that were found to significantly differentiate “baseline” and “asphyxia” states were lactate, succinate, lysine, fumarate, hypoxanthine and isoleucine (decrease) (*p* < 0.001). As far as the “baseline” against stabilization post-ROSC comparison is concerned, lactate, lysine, fumarate, hypoxanthine, succinate, acetate, alanine, glutamine, glutamate and choline differed significantly (*p* < 0.001). No metabolite survived False Discovery Rate correction and reached statistical significance in the direct “Asphyxia” versus “Resuscitation” comparison. **Conclusions**: This pilot study demonstrates that severe asphyxia in neonatal piglets is associated with a distinct serum metabolic signature, and several abnormalities remain detectable 30 min after ROSC, suggesting incomplete early metabolic recovery. The findings support the candidacy of lactate, succinate, fumarate, hypoxanthine and related metabolites for further assessment and validation as markers of acute hypoxic injury. Further investigation focused on these metabolites could also contribute to the elucidation of the involved pathophysiological mechanisms of PA and the development of novel therapeutic approaches.

## 1. Introduction

Perinatal asphyxia is a condition that can constitute a severe complication of the peripartum period leading to significant adverse sequelae both in the short and in the long term. According to the World Health Organization (WHO), it is defined as the failure to initiate and sustain breathing at birth and accounts for approximately one quarter of all neonatal deaths worldwide [1]. Several more definitions have been utilized in different studies and clinical settings. Overall, it is a condition characterized by a critical reduction in the supply of oxygenated blood to the fetus/neonate in the time period around delivery that can be the result of several maternal conditions and labor complications [2]. Most cases of perinatal asphyxia are attributed to intrapartum incidents. However, around 20% of the cases occur antepartum, and few cases might present in the early postnatal period [3].

Perinatal asphyxia accounts for approximately 900,000 newborn deaths every year according to the WHO, and it is one of the major causes of neonatal mortality and morbidity. Its incidence is estimated at approximately 1‰ to 6‰ live births [4]. However, this applies mainly to high-resource countries, while it is estimated to be 10 times higher in countries with more restricted healthcare access for mothers and neonates [3].

Perhaps the most severe and most well-studied complication of perinatal asphyxia is Hypoxic–Ischemic Encephalopathy (HIE). HIE is characterized as the neurological syndrome presenting in the neonate that has been exposed to different degrees of a hypoxic-–ischemic incident [5]. It is estimated to affect approximately 15 out of 10,000 live births [6] while the estimated incidence in low and middle-income countries is reported to be 10–20 per 1000 live births [7]. Furthermore, 15–20% of the affected newborns are reported to die in the early neonatal period, whereas 25–30% of the survivors present with significant neurological impairment [8]. Other than supportive care, the sole established treatment of HIE so far is therapeutic hypothermia (TH), as several neuroprotective agents are currently under investigation aiming at their utilization as adjuvant treatment [7,9].

Given the fact that it represents a state of generalized oxygen and nutrient deprivation to the tissues, perinatal asphyxia is known and expected to systematically affect the offspring and lead to several organ malfunction in the form of what is referred to as MOD (Multiple Organ Dysfunction). The involvement of different systems such as the cardiovascular, pulmonary, renal, gastrointestinal and endocrine, as well as the effect on liver, electrolyte balance and coagulation, have been described, and they can severely affect the mortality rate and the wellbeing of the affected neonate [2,4,10]. Furthermore, the potential implications of the above on the long-term prognosis are still not fully understood [11]. Subsequently, there is need for the discovery of novel biomarkers that can facilitate the diagnosis and classification of perinatal asphyxia as this could not only guide the further management and treatment of the patients but also allow for a more accurate and precise anticipation of the short- and long-term prognosis [6,12].

The term *metabolomics* refers to the scientific field that pertains to the study of small molecules, or metabolites, within the cells, biological systems and fluids [13]. These “metabolites” can be the substrates or products of metabolic pathways existing in all living systems, and their aggregate (namely the *metabolome*) can be considered as a form of molecular phenotype, filling the gap between the genotype and phenotype. Therefore, taking into consideration the fact that the organisms survive in a state of dynamic homeostasis, which is prone to changing in response to external and internal stimuli, alterations in the metabolic profile and pattern can be expected to reflect changes in pathways and processes in response to certain conditions such as disease, medication or diet [14,15]. Moreover, the fact that the methods applied in metabolomics allow for the simultaneous assessment of several endogenous and exogenous chemical entities provides us with a “snapshot” of the chemical identity of an organism at a certain time and in response to specific cellular processes [16]. Furthermore, the correlation of alterations of distinct metabolites with certain situations may lead to the discovery and application of novel biomarkers that could prove useful in the prognosis, diagnosis and management of certain medical conditions [17]. Among the analytical platforms employed in metabolomics, Nuclear Magnetic Resonance (NMR) spectroscopy occupies a prominent position, particularly for the profiling of biofluids such as serum and plasma [18]. Its inherent advantages include high reproducibility, non-selectivity—enabling the simultaneous detection of a broad range of metabolite classes including amino acids, organic acids, lipids, and carbohydrates—as well as its non-destructive nature and minimal sample preparation requirements [19]. Moreover, NMR metabolomics has the additional advantages of being non-biased and easily quantifiable; it is overall considered a fast technique, and it allows for identification of novel chemical substances [18,20].

As far as perinatal asphyxia and its complications are concerned, through the study of simultaneous dynamic alterations in the particles that constitute the metabolome and are measured in the various biological fluids, metabolomics can provide valuable insight in the early diagnosis and stratification of perinatal asphyxia, facilitate the detection of specific complications, and ideally provide us with means of assessing the response to treatment modalities and potentially future development as well [12].

The present study aimed to investigate the alterations in the serum metabolomic profile induced by perinatal asphyxia and subsequent resuscitation in a neonatal piglet model, with the objective of identifying specific metabolites that could serve as potential biomarkers for the early diagnosis and assessment of perinatal asphyxia and its associated complications.

## 2. Materials and Methods

A prospective, randomized experimental pilot animal study was designed. The study protocol was approved by the Greek General Directorate of Veterinary Services (Approval number: 1553/05/04/2018), and our study was conducted in accordance with the Greek legislation and the European Parliament Directives.

The current study was based on the protocol that has been described in our team’s previous publication [21]. The initial steps of the experimentation are briefly presented in the following paragraphs. Taking into consideration the fact that the first 4 days of life are considered part of the early neonatal period, which closely resembles transition at birth, the neonatal model that was used is expected to sufficiently simulate perinatal asphyxia. Hence, when the results of the current study are reported or analyzed, the term “perinatal asphyxia” will be utilized.

### 2.1. Animal Preparation

The study population comprised 33 female Landrace/Large White piglets, aged 1–4 days old with weight of 1300–2400 g, that were supplied by the same breeder (Validakis, registered breeder, Koropi, Greece) and transferred to the experimental facility (ELPEN’s Experimental, Research and Training Centre, European Ref No. EL 09 BIO 03) on the day of experimentation. Prior to the commencement of the experiment, all animals were examined by a veterinarian, and throughout the experimental procedure, all of the animals were treated in compliance with the Guide for the Care and Use of Laboratory animals. The population of the current study was the same that was utilized in our previous study, for which the female gender of the animals was a prerequisite. Since animal experimentation is characterized by the 3 Rs framework (*replacement*, *reduction*, *refinement*) [22], it was deemed appropriate to obtain the necessary samples from the animals that were already used.

The animals were initially sedated via intramuscular administration of ketamine hydrochloride (10 mg/kg) (Imalgène, Merial Laboratorios SA, Lyon, France), midazolam (0.5 mg/kg) (Dormicum, Roche, Athens, Greece) and atropine sulphate (0.01 mg/kg) (Atropine sulphate, Demo, Athens, Greece) as previously described [23]. Following that, general anesthesia was applied via intravascular administration of propofol (1 mg/kg) (Diprivan 1% *w*/*v*; Astra Zeneca, Luton, UK) and fentanyl (10 μg/kg) (Janssen Pharmaceutica, Beerse, Belgium). After that, the animals were intubated with a Portex cuffed endotracheal tube size 4 (Portex, 4.0 mm ID; Mallinckrodt Medical, Athlone, Ireland), and following the administration of fentanyl (20 μg/kg)—if required—and cis-atracurium (0.15 mg/kg) (Nimbex, 2 mg/mL; GlaxoSmithKline, Athens, Greece), they were placed on mechanical ventilation with the initial settings and target measurements that have been previously described [21].

Throughout the experimental procedure, the piglets remained under continuous monitoring of both their heart rate/ECG (leads I, II, III, avR, avL, avF) (Mennen Medical, Envoy; Papapostolou, Athens, Greece) and their oxygen saturation levels through a SpO2 sensor placed on their tongues. The animals’ temperature was also monitored using a rectal thermometer, and the target was 38 °C ± 1 °C.

The intravenous fluid and medication administration during the experimental procedure involved NaCl 0.9% and D/W 5%, as well as continuous intravenous propofol infusion (8–10 mg/kg/h) and bolus intravenous administration of fentanyl (10 μg/kg) and cis-atracurium (0.15 mg/kg) as needed in order to preserve sedation and anesthesia.

Finally, in order for the Systolic, Diastolic and Mean Arterial Pressure and Right Atrial Pressure to be continuously monitored, the animals’ common carotid and internal jugular veins were surgically revealed and catheterized with 3.5 Fr central catheters that were connected to electronic pressure transducers (3.5 Fr, USCI CR, Bart; Papapostolou).

Following catheter insertion and prior to the commencement of the experimental procedure, all animals were left to stabilize for 30 min. The desired hemodynamic parameters that were determined as consistent with “stabilization” are presented below:

○ Heart rate: 120–180 beats /min;
○ Systolic Arterial Blood Pressure: 70–90 mmHg;
○ Mean Arterial Blood Pressure: 60–80 mmHg;
○ Diastolic Arterial Blood Pressure: 40–60 mmHg;
○ Central Venous Pressure: 2–8 mm Hg;
○ SpO2: 90–95%.

Following stabilization, arterial blood gases as well as blood and urine samples were obtained from all the piglets (defined as “baseline” samples), and the main experiment commenced.

### 2.2. Experimental Protocol

Prior to any procedure, the study population was randomized into three categories with the use of a sealed envelope indicating the animals’ assignment to one of the three groups:

- Group A (12 piglets): control;
- Group B (11 piglets): perinatal asphyxia with no resuscitation;
- Group C (10 piglets): perinatal asphyxia with resuscitation.

Following randomization, only the primary investigator (E.-D.B.) was aware of the group allocation while the laboratory personnel, involved in the preparatory phase of the experiment, were blinded to further procedures. The primary investigator shared the necessary information with the experimental team at the indicated timepoints over the course of the experiment (induction of asphyxia, commencement of resuscitation). The initial step of data analysis was also blinded and, following retrieval of the primary results, the latter were correlated to the respective study groups and further processed.

Group A piglets remained under general anesthesia and mechanical ventilation for approximately one hour after stabilization, and following that, arterial blood gases and blood and urine samples were obtained (samples defined as “final”); the piglets were subsequently euthanized as has been previously described.

In the cases of Group B and C animals, their endotracheal tube (ETT) was occluded, thus leading to asphyxia, and intravenous infusion of propofol was ceased at the same time. The endpoint of asphyxia was the manifestation of hemodynamic compromise, defined as either bradycardia (HR < 60/min) or severe hypotension (MAP < 15 mmHg). The time interval between the occlusion of the endotracheal tube and the manifestation of hemodynamic compromise was recorded in all animals, and additional blood and urine samples were obtained (defined as “asphyxia”) as well as arterial blood gases that verified the presence of asphyxia. This was the experiment’s endpoint for Group B animals.

In the cases of Group C piglets, resuscitation commenced according to the ILCOR 2015 guidelines [24] after the clinical manifestation of asphyxia and confirmation of arterial blood gases. In brief, the resuscitation algorithm initially consisted of positive pressure ventilation with the use of Neopuff™ Infant Resuscitator (Fisher & Paykel Healthcare, Auckland, New Zealand) set at a Peak Inspiratory Pressure (PIP) of 30 cmH_2_O and a Positive End-Expiratory Pressure (PEEP) of 5 cmH_2_O. Gas flow supply was set at 8 L/min, and the FiO_2_ was the same that the animals were receiving prior to asphyxia. The response of the piglets was assessed both clinically and based on the hemodynamic parameters every 30 s as indicated by the NLS guidelines. In the absence of response, the algorithm proceeded to the administration of chest compressions, combined with positive ventilation. At this point, the administered FiO_2_ was increased to 100%. If there was still no improvement in the condition of the animals, a dose of intravenous adrenaline was given, while the combined administration of ventilation and chest compressions was maintained for cycles of 30 s. In the present study, the duration of resuscitation prior to achievement of return of spontaneous circulation (ROSC) ranged from 1 min to 2.5 min.

The time periods of asphyxia and resuscitation were recorded, as were the type and duration of resuscitation applied in each case and the administration of adrenaline, if indicated. ROSC was defined as the return of the hemodynamic parameters at the levels of the stabilization period with a deviation of ±10%. The endpoints of the experiment for Group C piglets were defined as either persistent asystole despite sufficient resuscitation efforts for 10 min, or return of spontaneous circulation (ROSC) and restoration of the hemodynamic parameters as previously mentioned, and the favorable or not outcome of resuscitation was recorded as well. Subsequently, the Group C piglets that were successfully resuscitated remained ventilated under sedation and intravenous fluid administration for 30 min with a target MAP of 50 mmHg; after that period of stabilization, blood and urine samples (characterized as “final” samples) and arterial blood gases were obtained. Following that, the animals were humanely euthanized. The obtainment of the “final” samples at only 30 min following ROSC, instead of a potentially longer time interval, was selected with the aim of investigating and focusing on the very early recovery window following resuscitation and stabilization rather than the long-term changes and the full recovery trajectory.

Strict adherence to resuscitation and post-resuscitation guidelines facilitated the minimization of treatment bias, and resuscitation was performed by qualified and sufficiently trained providers.

All of the blood samples were withdrawn through the central line catheters and placed in the indicated containers. At the end of the experiment, the blood samples were centrifuged at 3000 rpm for 15 min and the supernatant was stored in Cryo-vials at −80 °C until further processing.

As presented in Table 1, the blood sampling was performed at two timepoints in the cases of Groups A and B and at 3 timepoints in the cases of animals in Group C.

**Figure 1:**
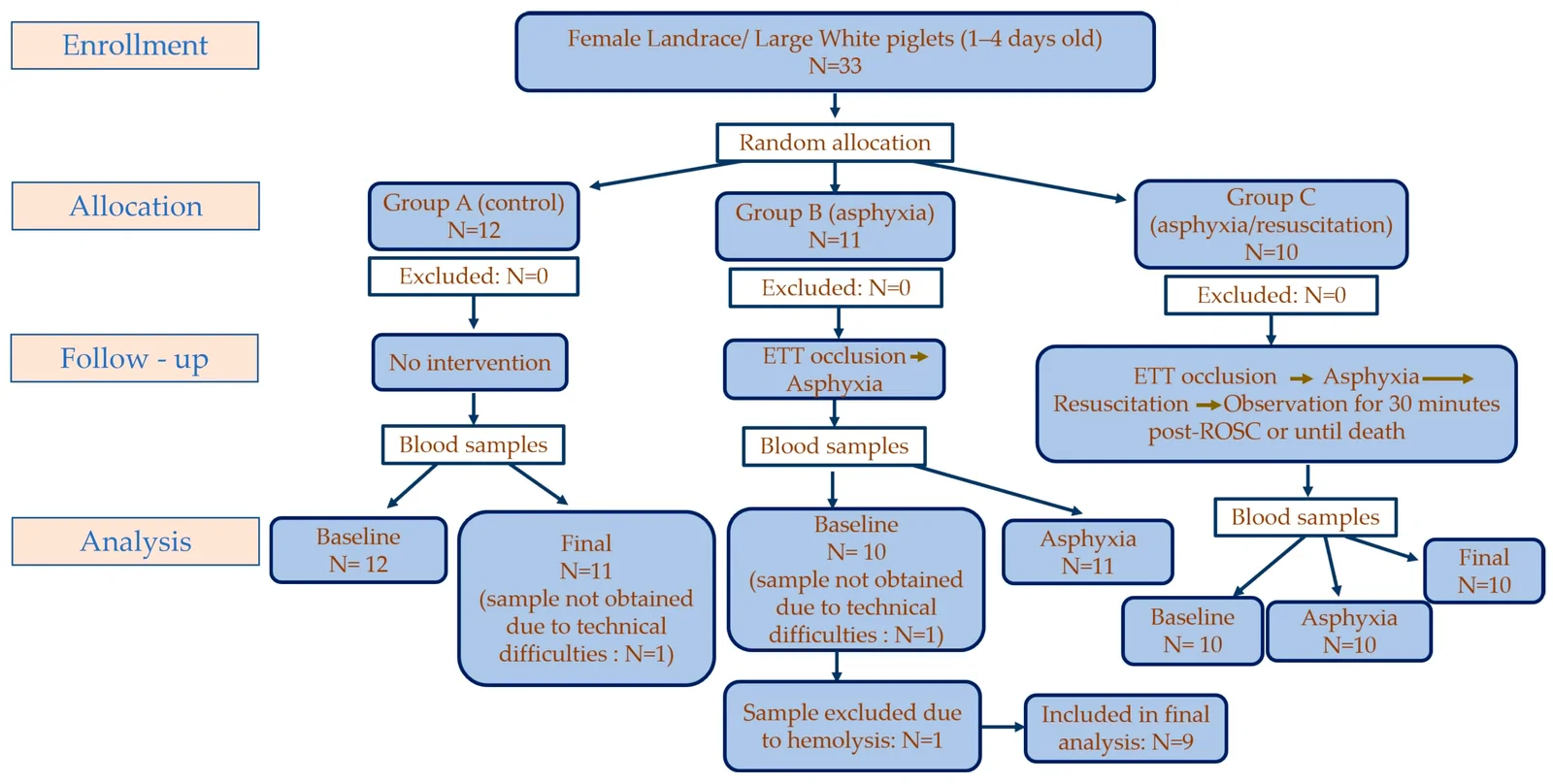
Flowchart presenting the steps of the experimental procedure and the obtainment of the blood samples that were included in the final analysis. ETT: endotracheal tube. ROSC: return of spontaneous circulation. “Baseline” sampling was performed 30 min after initial stabilization for all study groups, “asphyxia” samples were obtained following hemodynamic compromise from Group B and C animals and “final” samples were obtained approximately 1 h after baseline in Group A animals and after 30 min post-ROSC or prior to death in the cases of Group C animals.

In view of technical difficulties in obtaining blood samples at two timepoints in two different experiments, and following the exclusion of one blood sample due to hemolysis, the total number of serum samples that were processed was 73. More specifically, 31 of the samples were drawn at the “baseline” state, 11 samples were characterized as “final” deriving from “control” animals, 21 blood samples were obtained at the state of “asphyxia”, and 10 samples were characterized as “final” in the cases of animals that underwent asphyxia and were subsequently resuscitated and left to stabilize. The course of the experimental procedure and the sample obtainment at the different timepoints according to the experimental group is presented in Figure 1.

### 2.3. Sample Preparation

Serum samples were thawed at room temperature (25 °C) and extracted following the method by Moros et al. [25] and Nagana et al. [26]. In brief, 650 µL of serum was mixed with 1300 µL of methanol (1:2, *v*/*v*), vortexed, and incubated at −20 °C for 20 min. After centrifugation (14,000 rpm, 4 °C, 20 min) for protein precipitation, 1600 µL of supernatant was transferred and lyophilized to dryness (≈24 h; −110 °C condenser). Dried extracts were reconstituted in 540 µL of phosphate buffer in *D_2_O* (0.2 M; *Na_2_HPO_4_·2H_2_O/NaH_2_PO_4_*; pH 7.0), and 60 µL of TSP (5 mM) was added as an internal reference.

### 2.4. NMR Experimental Procedures

The NMR metabolic profiling spectra were acquired on a Bruker Ascend 500 MHz spectrometer (Bruker BioSpin AG, Billerica, MA, USA) equipped with a 5 mm double resonance broadband inverse (BBI) detection probe at the NMR Core Facility of the National and Kapodistrian University of Athens. Samples were analyzed on the NMR spectrometer in randomized run order, independent of experimental group assignment, minimizing the risk of batch effects or instrument drift confounding with group membership.

Experiments were acquired at 300 K, after a 5 min resting period for temperature stabilization, in automation mode, using a SampleCase-24 sample changer operated by IconNMR. Data acquisition and processing were performed with Bruker TopSpin 4.1.4 software. Metabolic profiling 1D NMR spectra were acquired using water suppression. 1D NOESY and T2-edited Carr–Purcell–Meiboom–Gill (CPMG) experiments were acquired with d1 = 5 s; AQ = 4.92 s; FID data points = 96 k; SW = 20 ppm; ns = 32 for noesygppr1d and 96 for cpmgpr1d, respectively. The transmitter offset was set manually to achieve optimal suppression of the residual water signal for both experiments. FIDs were zero-filled and multiplied by an exponential weighting function corresponding to a line broadening of 0.3 Hz before Fourier transformation. Chemical shift values were referenced to the residual TSP signal (0.00 ppm) which was used as internal standard. In a selected sample, a J-resolved and 2D TOCSY and HSQC experiments were acquired for metabolite identification. Metabolite identification was assisted using MetaboMiner with 2D TOCSY and HSQC-DEPT spectra acquired for selected samples [27]. Peak-picking lists were uploaded to MetaboMiner for candidate screening and refined by spectral overlay.

More specifically, the above validation NMR experiments were acquired using phase sensitive MLEV sequence with d1 = 2 s; FID data points = 4 k (F2) and 320 (F1); SW = 20 ppm; and ns = 48 and mixing time (d9) = 0.08 s with suppression of the residual water signal. Furthermore, phase-sensitive HSQC-DEPT experiments were performed using Echo/Antiecho-TPPI gradient selection with decoupling during acquisition (hsqcedetgpsisp2.3), with FID data points = 4 k (F2) and 288 (F1); SW = 12 ppm (F2) and 180 (F1); and ns = 160 in non-uniform sampling mode acquisition with 50% amount of sparse sampling. Finally, the .zip archive containing all files was uploaded to the NMRProcFlow open-access web tool to carry out chemical shift calibration, baseline correction, S/N ratio identification, along with alignment, normalization and bucketing. For alignment, an interactive approach was conducted, indicating that each interval was selected individually using CluPA and the least squares method. The interactive spectral alignment and bucket selection was performed by one operator (P.C.) who was blinded to experimental group allocation at the time of processing, in order to minimize the risk of selection bias during this step. Bucket selection was independently cross-checked by a second evaluator (A.C.), also blinded to group allocation, to verify reproducibility of this preprocessing step.

All spectra underwent normalization via the Probabilistic Quotient Normalization (PQN) technique, and an advanced intelligent bucketing module was applied in the 0.8–8.5 spectra range.

### 2.5. Statistical Analysis

All statistical analyses were performed using MetaboAnalyst 6.0. For univariate comparisons, non-parametric Wilcoxon signed-rank (paired) tests were used throughout, matching samples from the same animal across timepoints for each comparison. To account for multiple testing, *p*-values were adjusted using the False Discovery Rate (FDR) method (Benjamini–Hochberg), and results were considered statistically significant at *p* < 0.05 and *q* < 0.05.

Multivariate analysis was conducted to explore metabolic differences between experimental groups. Principal Component Analysis (PCA) was applied as an unsupervised method for pattern recognition and outlier detection, while Orthogonal Partial Least Squares Discriminant Analysis (OPLS-DA) was used as a supervised approach to maximize group separation. Model performance and robustness were evaluated using R^2^ and Q^2^ metrics, along with permutation testing (*n* = 1000). A VIP score threshold of >1.0 was applied to define top discriminant metabolites in OPLS-DA models.

Receiver Operating Characteristic (ROC) curve analysis was employed to feature selection based on Area Under the Curve (AUROC > 0.75) and statistical significance (*p* < 0.05).

Metabolic pathway analysis was performed separately for the metabolite sets identified as significant in each discriminant comparison (“Asphyxia” vs. “Baseline” and “Baseline” vs. “Resuscitation”), using the Pathway Analysis module of MetaboAnalyst 6.0. Over-representation analysis (hypergeometric test) was combined with topology analysis (relative-betweenness centrality) using the Sus scrofa (pig) specific KEGG pathway library. Pathway impact and statistical significance (raw *p*, Holm-adjusted *p*, and FDR) were calculated for each matched pathway.

Age and body weight were compared between groups using the Mann–Whitney U test to assess their potential as confounding variables. Also, the relationship between asphyxia duration and the concentration of key discriminant metabolites was assessed using Spearman’s rank correlation coefficient. Cumulative anesthetic doses (propofol, fentanyl, cis-atracurium) were compared between groups using the Mann–Whitney U test and correlated with key discriminant metabolites using Spearman’s rank correlation.

Achieved statistical power was calculated for the three metabolites most consistently identified as discriminant across both comparisons (lactate, succinate, hypoxanthine). Power was computed using Cohen’s *d*, derived from the observed group means and standard deviations of each metabolite’s raw NMR-derived intensity, applied to a two-independent-samples t-test framework (*α* = 0.05, two-tailed), using the actual sample sizes of each comparison (Asphyxia *n* = 21 vs. Baseline *n* = 42; Baseline *n* = 42 vs. Resuscitation *n* = 10). This approach avoids the circularity inherent in deriving power estimates directly from the fitted OPLS-DA model’s own R^2^Y/Q^2^ statistics, which—being supervised, overfitting-prone metrics optimized to maximize class separation—cannot serve as an independent estimate of the true population effect size.

Supplementary statistical analyses, including Spearman rank correlation between asphyxia duration and metabolite concentrations, retrospective power calculations (Cohen’s *d*), descriptive summary statistics, and paired Wilcoxon signed-rank tests for the descriptive comparisons, were performed in Python 3.12 using pandas (3.0), SciPy (1.17), and statsmodels (0.14) and are reported alongside the corresponding MetaboAnalyst 6.0 outputs in the Section 3. The 95% confidence intervals for AUROC values were estimated via bootstrap resampling (2000 iterations, percentile method) using scikit-learn.

It is worth pointing out that this is a pilot-scale study and the small sample size constitutes a limitation. As a result, there might be a decrease in the power of the study, leading to the failure to trace subtle changes. Therefore, the results should be interpreted with caution.

## 3. Results

### 3.1. Exploratory Analysis

Prior to performing discriminant analysis, an exploratory statistical evaluation was conducted to determine whether the control samples obtained at “baseline” and at the “final” timepoint could be considered as a single group. To this end, a series of univariate t-tests was applied across all detected metabolic features to compare the metabolic profiles between the control-“baseline” and control-“final” conditions.

**Figure 2:**
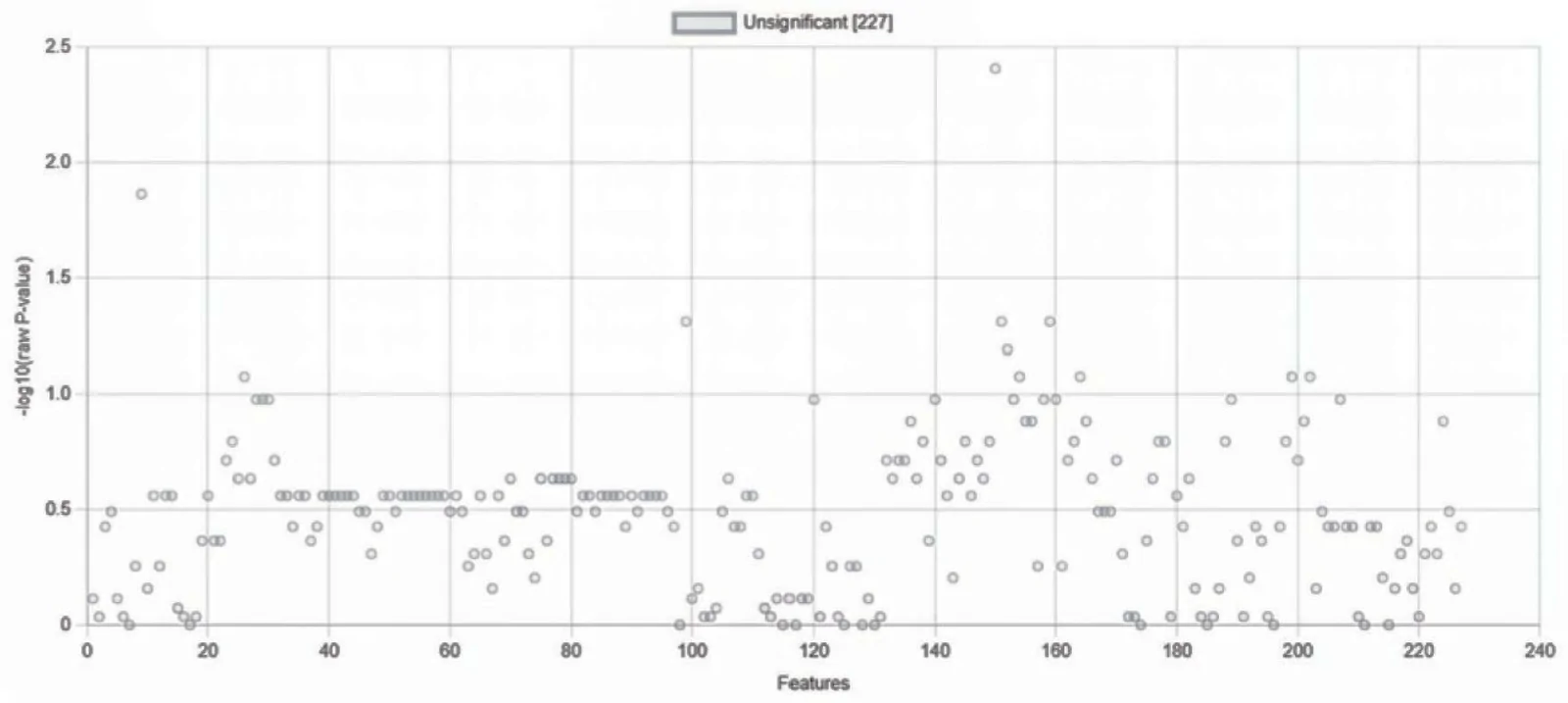
Volcano plot illustrating the statistical comparison between control-“baseline” and control-“final” serum samples across 227 metabolic features. No features exhibited statistically significant differences after False Discovery Rate (FDR) correction (*q* > 0.05), supporting the decision to merge the two control timepoints into a single unified group for further analysis.

As shown in Figure 2, none of the 227 evaluated features (obtained from total NMR spectra) exhibited statistically significant differences after testing correction (FDR-adjusted *q*-value > 0.05 for all features). The corresponding volcano plot demonstrates that all variables fall below the commonly accepted threshold for statistical significance (−log10(*q*) < 1.3), indicating a high degree of consistency between the two timepoints in the control group. Nevertheless, although the volcano plot showed no metabolites with significant alteration, minor time trends could exist. Merging the control timepoints streamlines analysis but may mask very small temporal shifts.

Based on these findings, it was deemed appropriate to merge the “baseline” and control-“final” samples into a unified control group for subsequent multivariate and discriminant analyses. This consolidation enhances the statistical power for downstream comparisons without introducing timepoint bias within the control population. This unified group, consisting of the “baseline” samples obtained from all three animal groups and the “final” samples obtained from the control group (A) will be addressed to as “baseline” from this point onwards. Furthermore, since the experimental protocol was the same for Group B and Group C animals until the timepoint of asphyxia, the “asphyxia” sample pool consisted of samples deriving from both animal groups. Following the adjustment, the sample groups were formed as follows: the unified “baseline” group consisted of 42 samples, the “asphyxia” group involved 21 samples and the “final” post-resuscitation group consisted of 10 samples. Subsequently, comparisons were performed among the three different sample groups (“baseline” versus “asphyxia”, “asphyxia” versus “post-resuscitation” and “baseline” versus “post-resuscitation”) as described in the following sections. The samples that were obtained from Group C animals after the period of stabilization post-resuscitation reflect the metabolomic profile post-ROSC and will be referred to as “resuscitation” samples over the statistical analysis.

**Figure 3:**
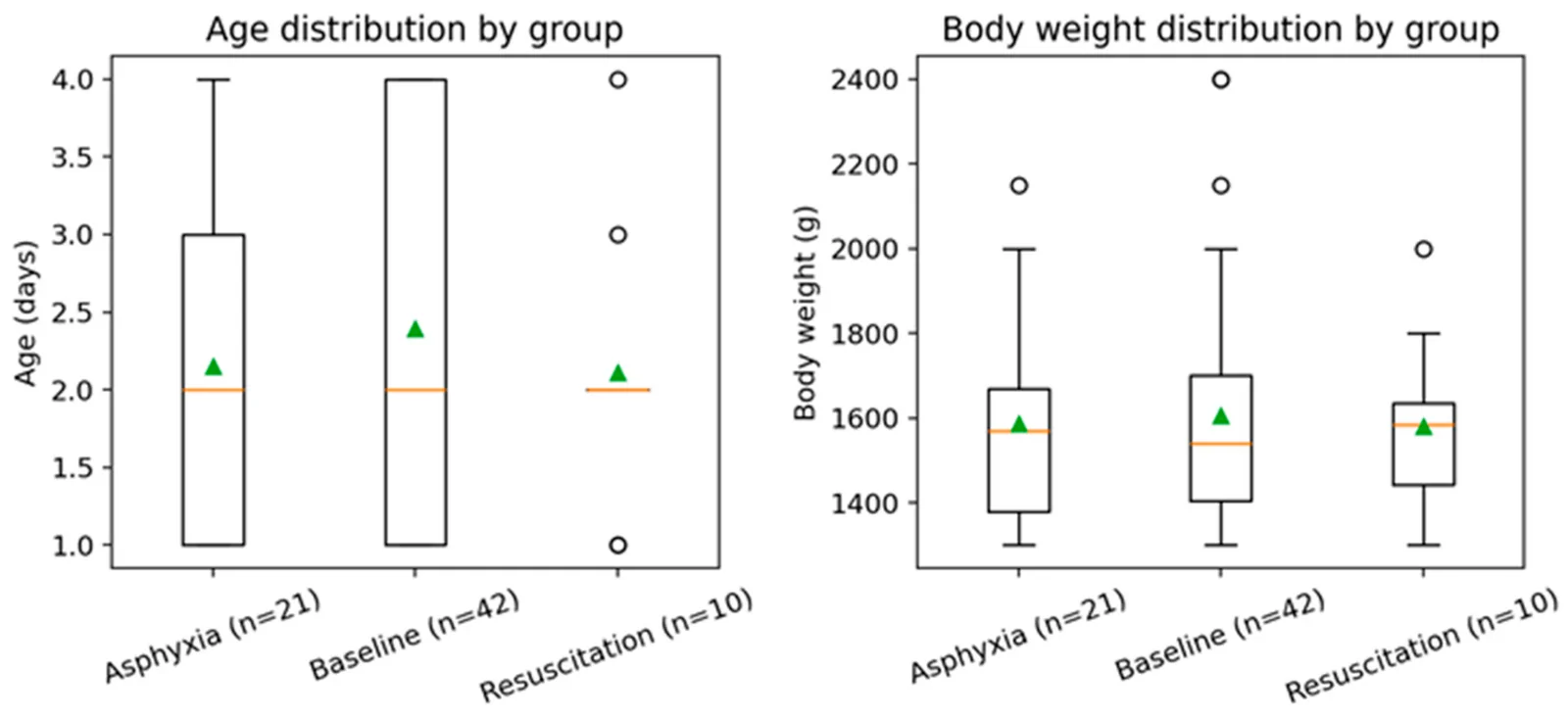
Age (**left**) and body weight (**right**) distributions by group. Boxes show median and Interquartile Range; markers indicate group means. Mann–Whitney U tests showed no significant between-group differences for either variable (all *p* > 0.48), indicating that both are unlikely to confound the reported metabolic signatures.

Piglet age at the time of sampling did not differ significantly between groups (“Asphyxia”: 2.15 ± 1.14 days vs. “Baseline”: 2.39 ± 1.22 days, Mann–Whitney *p* = 0.487; “Baseline” vs. “Resuscitation”: 2.39 ± 1.22 vs. 2.11 ± 0.93 days, *p* = 0.669), indicating that age is unlikely to have confounded the observed metabolic differences (Figure 3, left panel). Similarly, body weight did not differ significantly between groups (“Asphyxia”: 1587 ± 235 g vs. “Baseline”: 1606 ± 271 g, *p* = 0.896; “Baseline” vs. “Resuscitation”: 1606 ± 271 g vs. 1580 ± 207 g, *p* = 1.000), indicating that body weight is unlikely to have confounded the metabolic comparisons (Figure 3, right panel).

**Figure 4:**
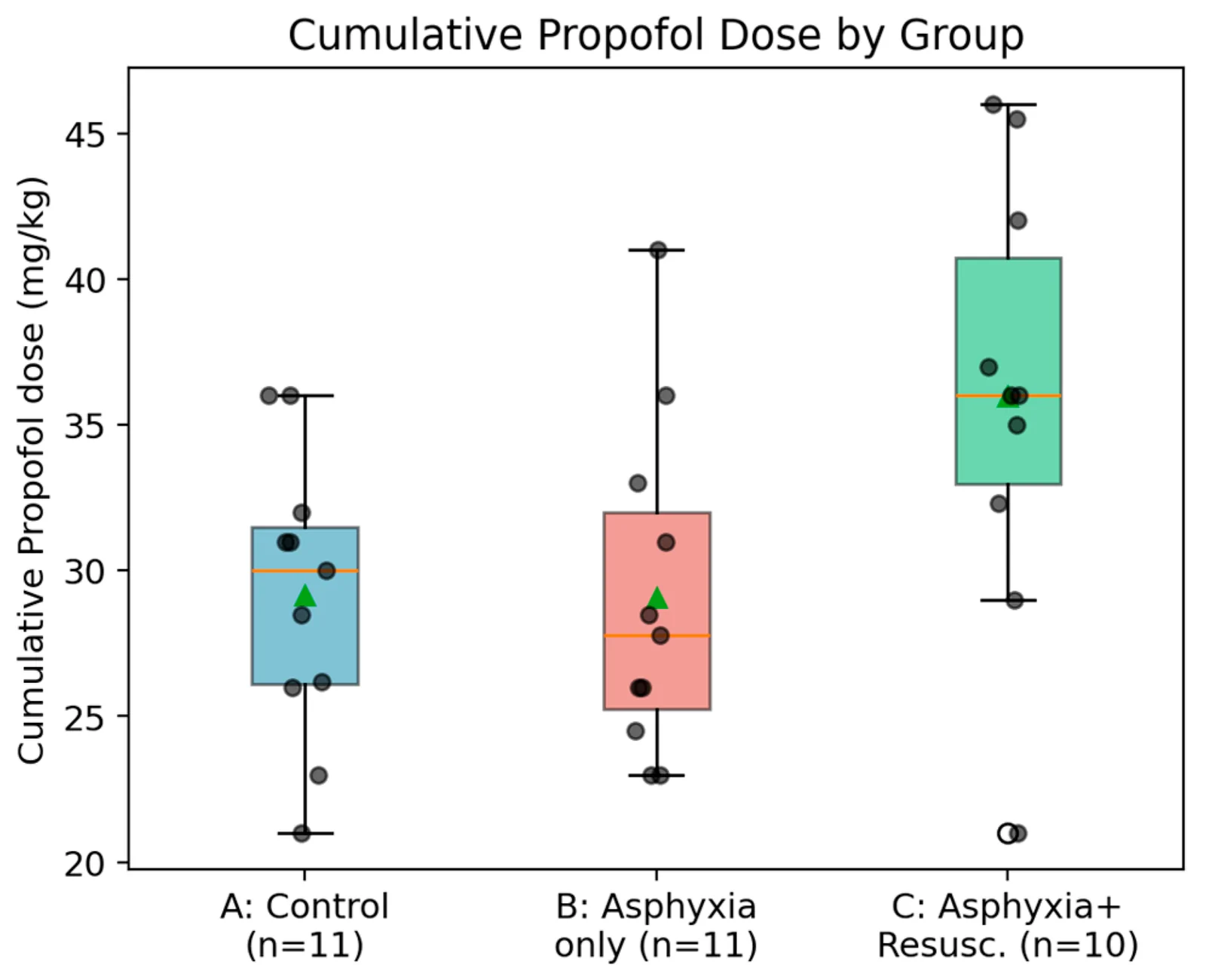
Cumulative propofol dose (mg/kg) each animal received by animal group. Boxes show median and IQR; individual animals overlaid (jittered). IQR: Interquartile Range.

**Figure 5:**
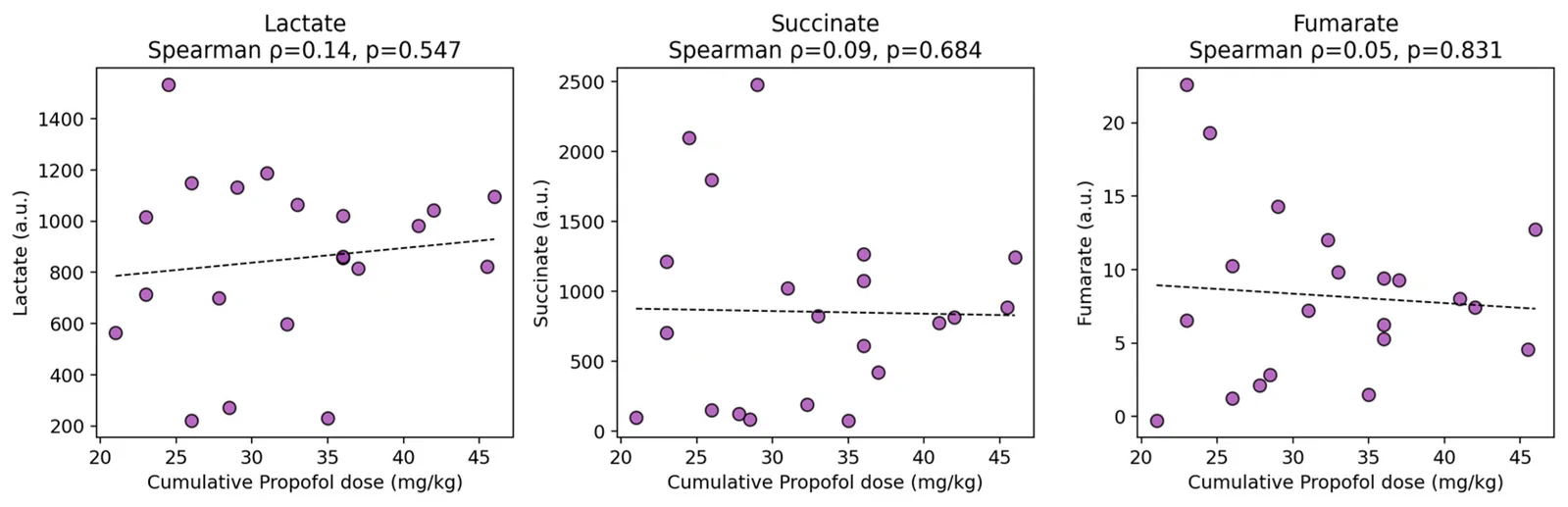
Spearman correlation between cumulative propofol dose (mg/kg) and key discriminant metabolites within the “asphyxia” group (*n* = 21). Dashed lines show linear trend for visualization.

Cumulative anesthetic doses that each animal received throughout the experiment were compared between the animal groups (Table 2, Figure 4). No precise data regarding the exact doses were available for one of the animals in the control group (A). Propofol dose was significantly higher in Group C (asphyxia with resuscitation; median 36.0 mg/kg) compared to both Group B (asphyxia without resuscitation; median 27.8 mg/kg, *p* = 0.031) and Group A (control; median 30.0 mg/kg, *p* = 0.024), reflecting additional propofol administered during the period of stabilization post-resuscitation. Fentanyl and cis-atracurium doses did not differ significantly between groups (*p* > 0.1). To assess whether this differential propofol exposure could account for the reported metabolic signature, propofol dose was correlated with the three metabolites of greatest mechanistic concern (lactate, succinate, fumarate) within the “asphyxia” group. No significant correlations were observed (lactate: *rho* = 0.139, *p* = 0.547; succinate: *rho* = 0.094, *p* = 0.684; fumarate: *rho* = 0.049, *p* = 0.831; Figure 5), indicating that propofol dose is not a meaningful driver of the reported metabolite levels in this cohort.

### 3.2. Multivariate Analysis

**Figure 6:**
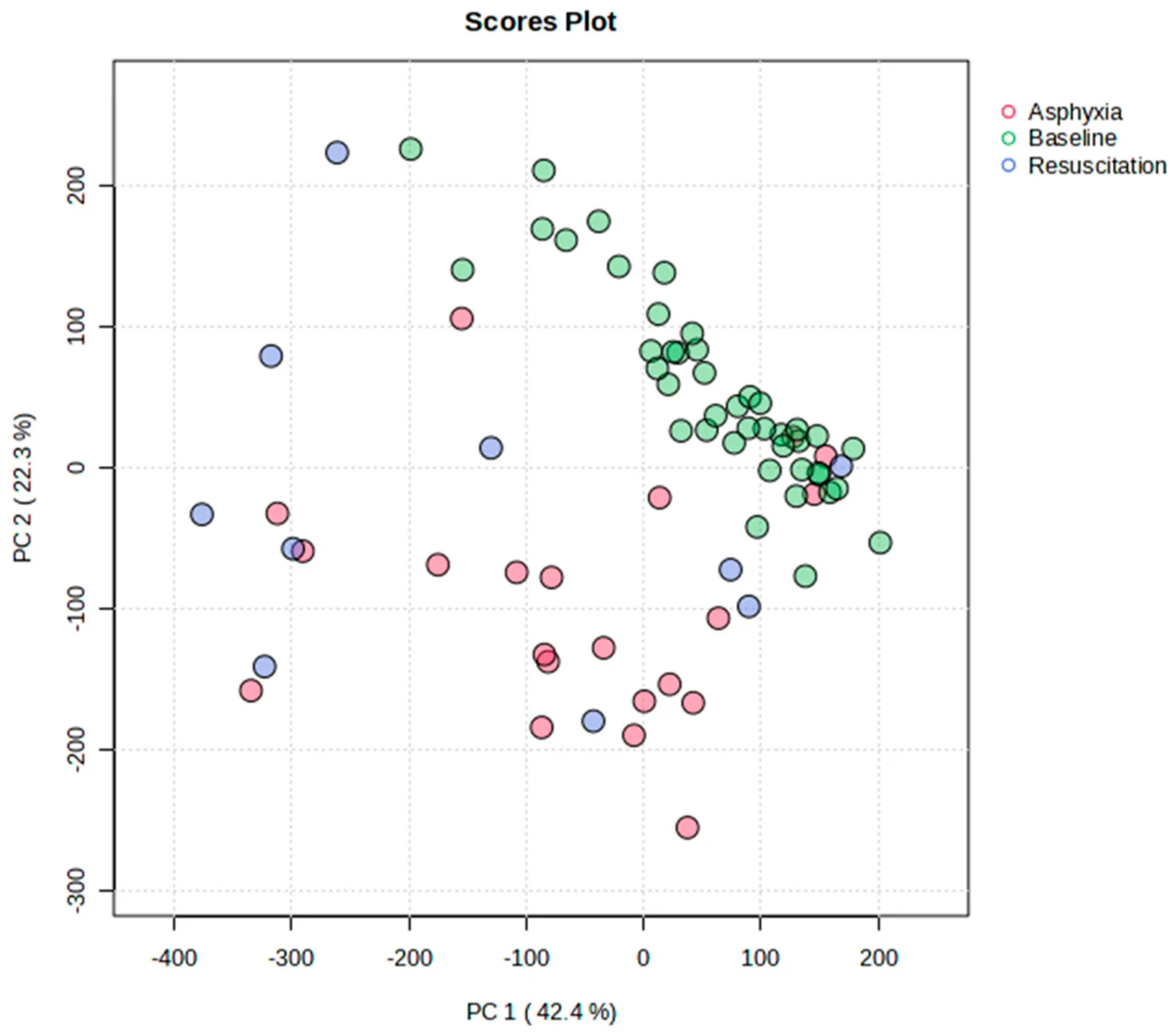
PCA scores plot based on untargeted serum metabolomic profiles of piglets across three experimental conditions: “Baseline” (green), “Asphyxia” (red), and “Resuscitation” (blue). Principal components 1 and 2 explain 42.4% and 22.3% of the variance, respectively. Clear group separation supports condition-specific metabolic responses. PCA: Principal Component Analysis.

To further explore the overall variation in serum metabolomic profiles across experimental conditions, an unsupervised multivariate analysis was conducted using Principal Component Analysis (PCA). The PCA scores plot (Figure 6) reveals clear clustering and separation of samples corresponding to the three distinct groups: “Baseline” (control), “Asphyxia”, and “Resuscitation”.

Principal Component 1 (PC1) and Principal Component 2 (PC2) accounted for 42.4% and 22.3% of the total variance, respectively, cumulatively explaining 64.7% of the dataset’s variance. The “baseline” samples clustered tightly together, indicating a homogeneous metabolic profile in the absence of any hypoxic insult. In contrast, the “asphyxia” group formed a distinct cluster along PC1, suggesting a marked shift in the metabolic phenotype under hypoxic conditions. The “resuscitation” group appeared to shift partially back towards the “baseline” cluster but remained metabolically distinct from both “baseline” and “asphyxia” groups, indicating incomplete restoration of metabolic homeostasis 30 min post-return of spontaneous circulation.

These findings support the hypothesis that perinatal asphyxia induces profound metabolic perturbations, which persist despite short-term resuscitation. PCA thus provides an initial overview of group-level metabolic discrimination and informed subsequent supervised multivariate modeling and univariate investigations.

### 3.3. Bucket Reduction Process for Biomarker Discovery

**Figure 7:**
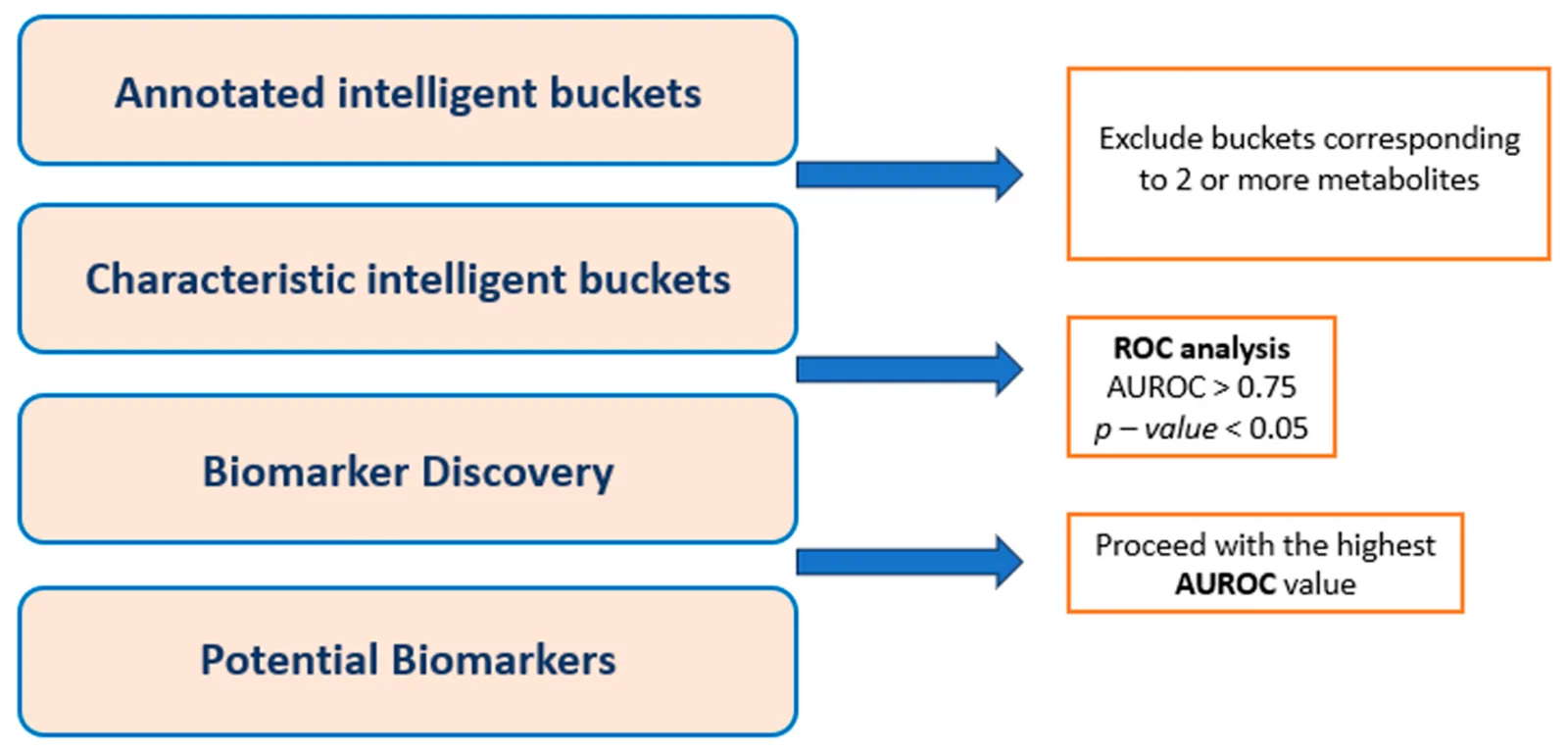
Stepwise bucketing-based feature reduction workflow used for biomarker discovery. Annotated intelligent buckets were filtered to retain only characteristic, non-overlapping features. Subsequent ROC analysis (AUROC > 0.75, *p* < 0.05) enabled prioritization of discriminant buckets, leading to the identification of potential biomarkers. AUROC: Area Under the Receiver Operating Characteristic curve.

To identify discriminant metabolic features associated with perinatal asphyxia and its recovery phase, we adopted a structured biomarker discovery pipeline grounded in our previously validated methodology [28]. This approach integrates intelligent bucketing, feature refinement, and ROC-based filtering to ensure the biological relevance and diagnostic potential of selected metabolites (Figure 7).

Initially, all pre-processed NMR features were grouped into annotated intelligent buckets, each representing a distinct resonance signal. To reduce redundancy and ambiguity, buckets corresponding to overlapping or co-eluting metabolites were excluded, yielding a refined set of characteristic intelligent buckets. These buckets served as input variables for subsequent statistical screening.

ROC analysis was then applied to evaluate the discriminant capacity of each feature separately for each pairwise comparison (“Baseline” vs. “Asphyxia”; “Baseline” vs. “Resuscitation”). The same AUROC > 0.75 and *p* < 0.05 dual threshold was applied independently within each comparison for feature retention. Only those features with an area under the ROC curve (AUROC) greater than 0.75 and *p*-value below 0.05 were retained for further analysis. This dual threshold ensured both statistical and clinical relevance.

From this process, a subset of potential biomarkers was identified, reflecting those metabolic features most strongly associated with the pathophysiological state of asphyxia and/or the response to resuscitation. Features with the highest AUROC values were prioritized for further biological interpretation, multivariate modeling, and pathway-level insight.

To assess the precision of the reported discriminative performance, 95% confidence intervals for the AUROC of the key biomarkers were estimated via bootstrap resampling (2000 iterations). For the “Asphyxia”-vs.-“Baseline” comparison, confidence intervals confirmed strong discriminative performance (Table 3). For the “Baseline”-vs.-“Resuscitation” comparison, AUROC values were similarly high, though with wider confidence intervals reflecting the smaller Resuscitation group size (*n* = 10), (Table 4). All lower confidence bounds remained close to or above the 0.75 selection threshold used for biomarker retention, supporting the robustness of the biomarker selection process despite the modest sample sizes of this pilot study.

The consistent application of this methodology supports the robustness and translational potential of the discovered biomarkers.

### 3.4. Discriminant Analysis Between Asphyxia and Baseline

**Figure 8:**
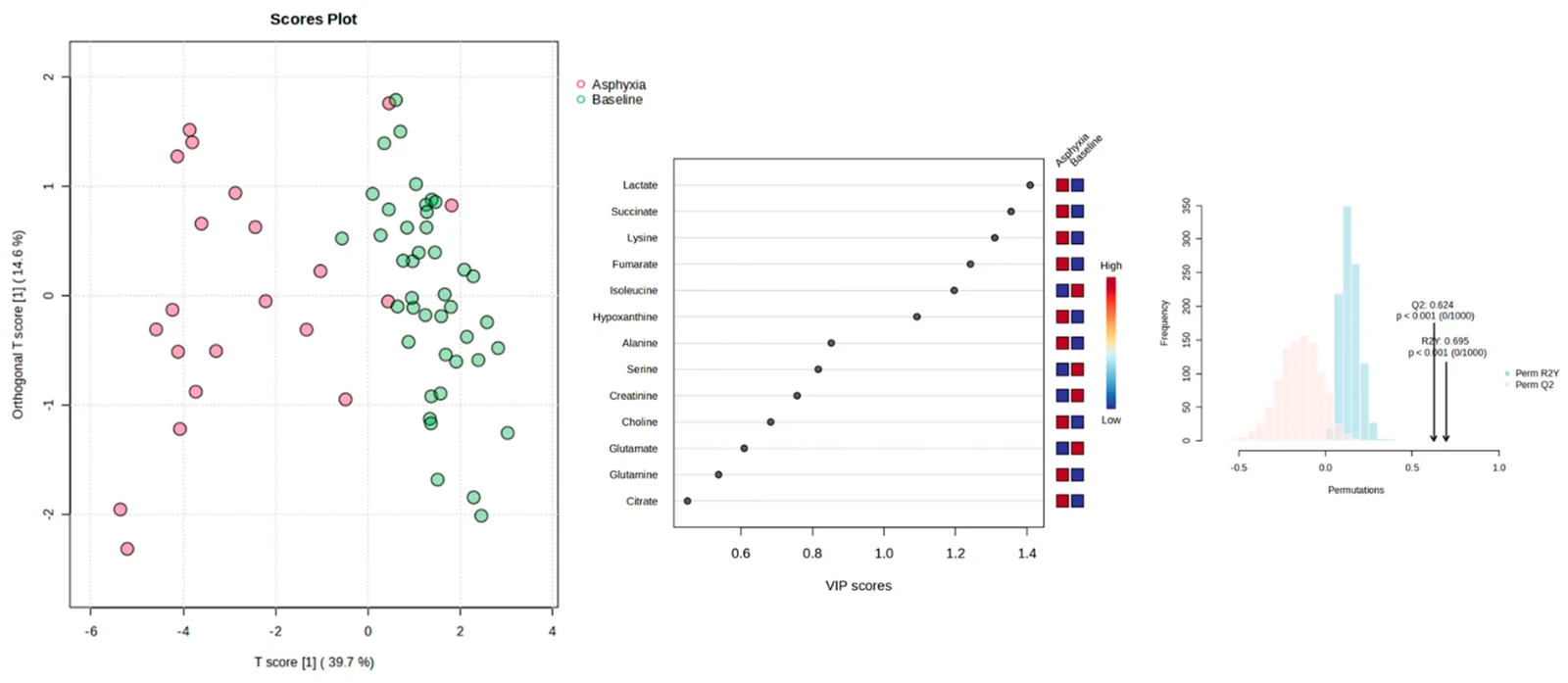
Discriminant analysis between “baseline” and “asphyxia” groups. **Left**: OPLS-DA scores plot showing clear separation between “baseline” (green) and “asphyxia” (red) piglet serum samples. **Center**: VIP scores identifying the top metabolites contributing to class discrimination, with lactate, succinate, lysine, fumarate, isoleucine and hypoxanthine ranked highest. **Right**: Permutation test validating model performance (R^2^Y = 0.695, Q^2^ = 0.624, *p* < 0.001). OPLS-DA: Orthogonal Partial Least Squares Discriminant Analysis, VIP: Variable Importance in Projection. Group colors are independently assigned by the analysis software for each model and do not correspond across figures; readers should refer to the legend within each panel.

To investigate the metabolic alterations induced by perinatal asphyxia, a supervised multivariate model was built using Orthogonal Partial Least Squares Discriminant Analysis (OPLS-DA), comparing “baseline” and “asphyxia” serum metabolomic profiles. This analysis was performed using the reduced set of intelligent buckets derived from our biomarker discovery pipeline (see Figure 8).

The resulting OPLS-DA scores plot (Figure 8, left) shows a clear separation between the two groups along the predictive component (T [1], 39.7% of variance), while the orthogonal component (To [1], 14.6%) captures variation unrelated to class separation. This distinct clustering confirms that perinatal asphyxia induces a significant and consistent shift in the serum metabolome in comparison to the baseline state.

Model robustness was confirmed through 1000-fold permutation testing (Figure 8, right), yielding high R^2^Y (0.695) and Q^2^ (0.624) values with associated *p*-values < 0.001, indicating that the model was not overfitted and had strong predictive capability.

Further insight was gained through Variable Importance in Projection (VIP) scoring (Figure 8, center), which identified several key metabolites contributing to class separation. The top discriminant features included lactate, succinate, lysine, fumarate, isoleucine, and hypoxanthine, all of which are known to be involved in hypoxia-related energy disruption and oxidative stress pathways. These metabolites not only exhibited high VIP values (>1.0) but also showed consistent group-wise intensity trends, supporting their biological relevance.

**Figure 9:**
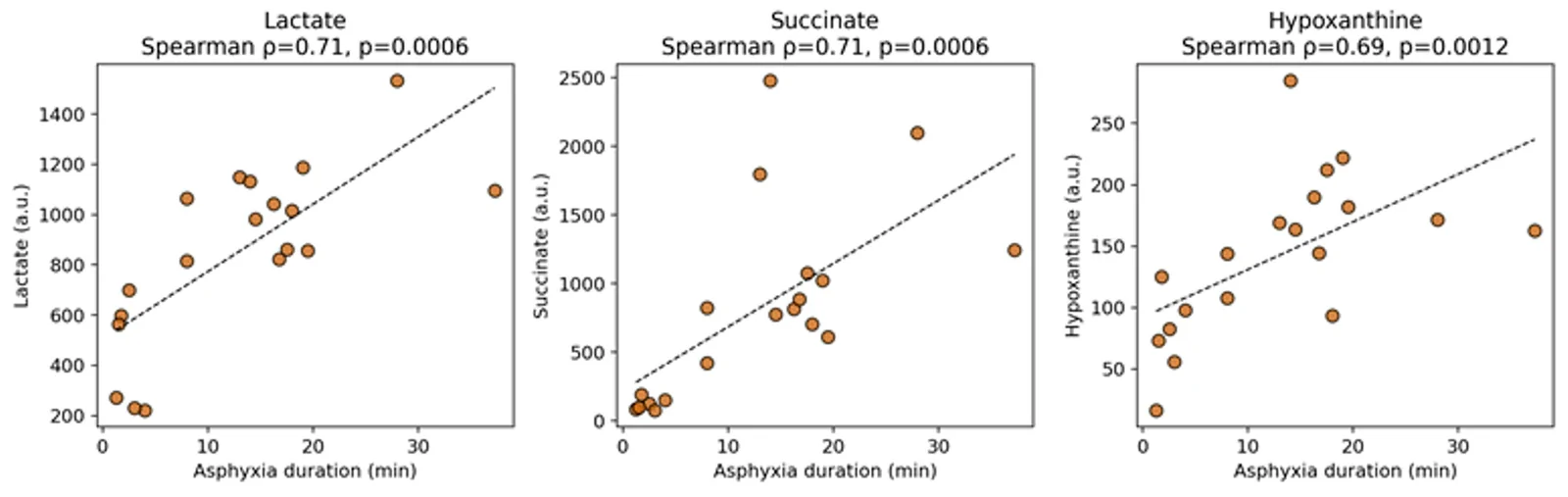
Spearman correlations between individual asphyxia duration (minutes) and metabolite concentration (arbitrary units) for lactate, succinate, and hypoxanthine in the asphyxia group (*n* = 21, *n* = 19 with recorded duration). Dashed lines show the linear trend for visualization; correlation statistics are rank-based (Spearman *ρ*).

To assess whether the severity of the metabolic response scaled with the duration of the hypoxic insult, Spearman correlations were calculated between individual asphyxia duration (range 1.25–37.25 min) and the concentration of the three metabolites most strongly associated with the asphyxia signature. Lactate (*ρ* = 0.712, *p* = 0.0006), succinate (*ρ* = 0.713, *p* = 0.0006), and hypoxanthine (*ρ* = 0.686, *p* = 0.0012) all showed strong, statistically significant positive correlations with asphyxia duration, indicating a dose-dependent relationship between the severity/length of the hypoxic–ischemic insult and the magnitude of the metabolic derangement (Figure 9). Fumarate showed a weaker, borderline association (*ρ* = 0.441, *p* = 0.059).

### 3.5. Discriminant Analysis Between Baseline and Resuscitation

To assess the degree of metabolic restoration following perinatal asphyxia and resuscitation, an OPLS-DA model was constructed to discriminate between serum profiles at “baseline” and 30 min post-ROSC. This model utilized the same reduced feature set derived from intelligent bucketing and biomarker preselection criteria described previously.

**Figure 10:**
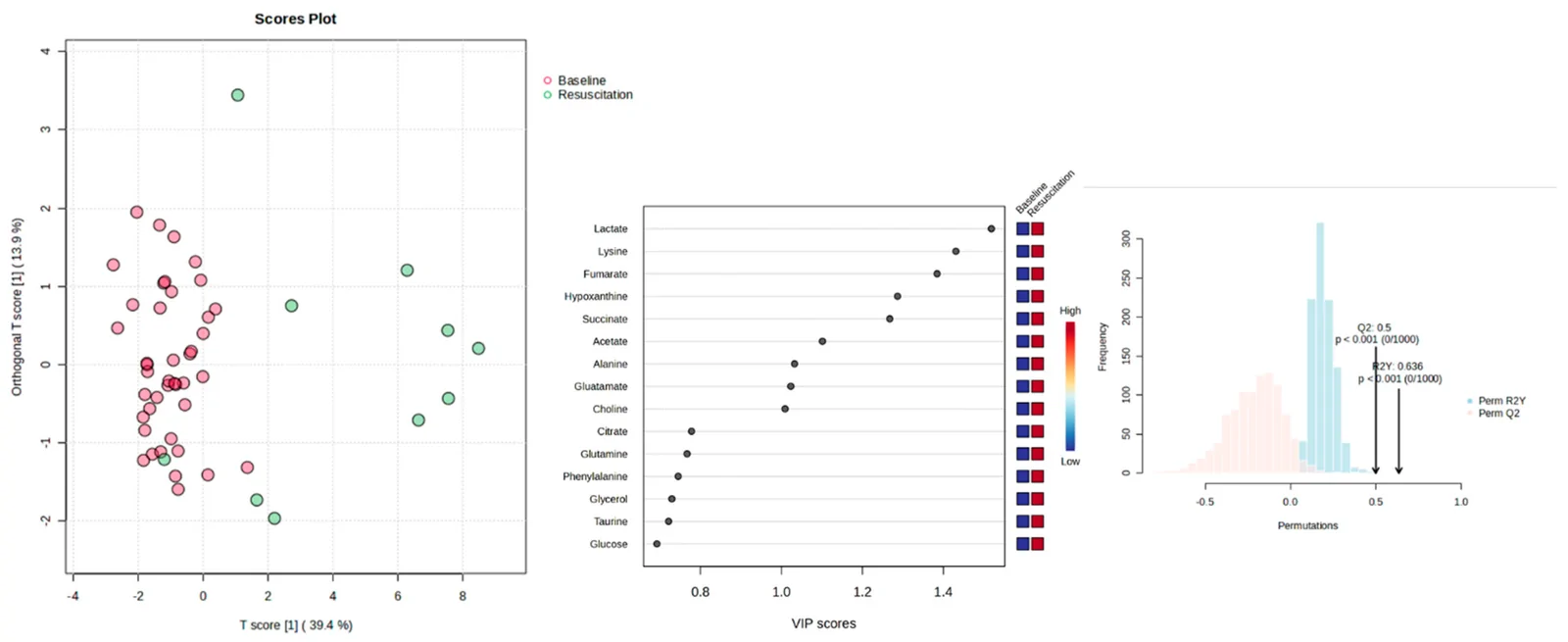
OPLS-DA model comparing “baseline” and “post-resuscitation” metabolomic profiles. Left: OPLS-DA score plot shows moderate separation between “baseline” (red) and “resuscitation” (green) samples. Center: VIP scores highlighting the most influential metabolites for class discrimination, including lactate, lysine, fumarate, hypoxanthine, succinate, acetate, alanine, glutamate and choline. Right: Permutation test confirms model robustness (R^2^Y = 0.636, Q^2^ = 0.500, *p* < 0.001). OPLS-DA: Orthogonal Partial Least Squares Discriminant Analysis, VIP: Variable Importance in Projection. Group colors are independently assigned by the analysis software for each model and do not correspond across figures; readers should refer to the legend within each panel.

The OPLS-DA score plot (Figure 10, left) demonstrated partial group separation, indicating that although resuscitated animals began shifting back toward “baseline” metabolic states, distinct differences still persisted. The first predictive component (T [1]) explained 39.4% of the variation, while the orthogonal component captured an additional 13.9% of the total variance.

Model validation using 1000-fold permutation testing (Figure 10, right) supported the statistical robustness of the model, with R^2^Y = 0.636 and Q^2^ = 0.500, both statistically significant (*p* < 0.001). These values indicate moderate-to-strong class predictability and model reliability.

The VIP score plot (Figure 10, center) identified the top discriminant metabolites contributing to group separation. These included lactate, lysine, fumarate, hypoxanthine, succinate, acetate, alanine, glutamate and choline, all of which are well-established metabolic markers of hypoxia, mitochondrial dysfunction, or impaired energy metabolism. Importantly, several of these metabolites—such as lactate and succinate—remained elevated even after resuscitation, reinforcing the observation that complete metabolic recovery was not achieved within the early post-ROSC timeframe.

Overall, the model suggests that although resuscitation alters the metabolic profile relative to the “asphyxia” phase, the metabolome does not return to its “baseline” state within 30 min. These persistent biochemical changes may reflect residual oxidative stress, incomplete mitochondrial recovery, or post-ischemic tissue dysfunction.

### 3.6. Univariate Analysis: Volcano Plot Interpretation

To complement multivariate modeling and gain insight into individual metabolite behavior, univariate statistical analysis was performed for all pairwise group comparisons using volcano plots, integrating both fold-change (log_2_FC) and statistical significance (−log_10_ *p*-value).

**Figure 11:**
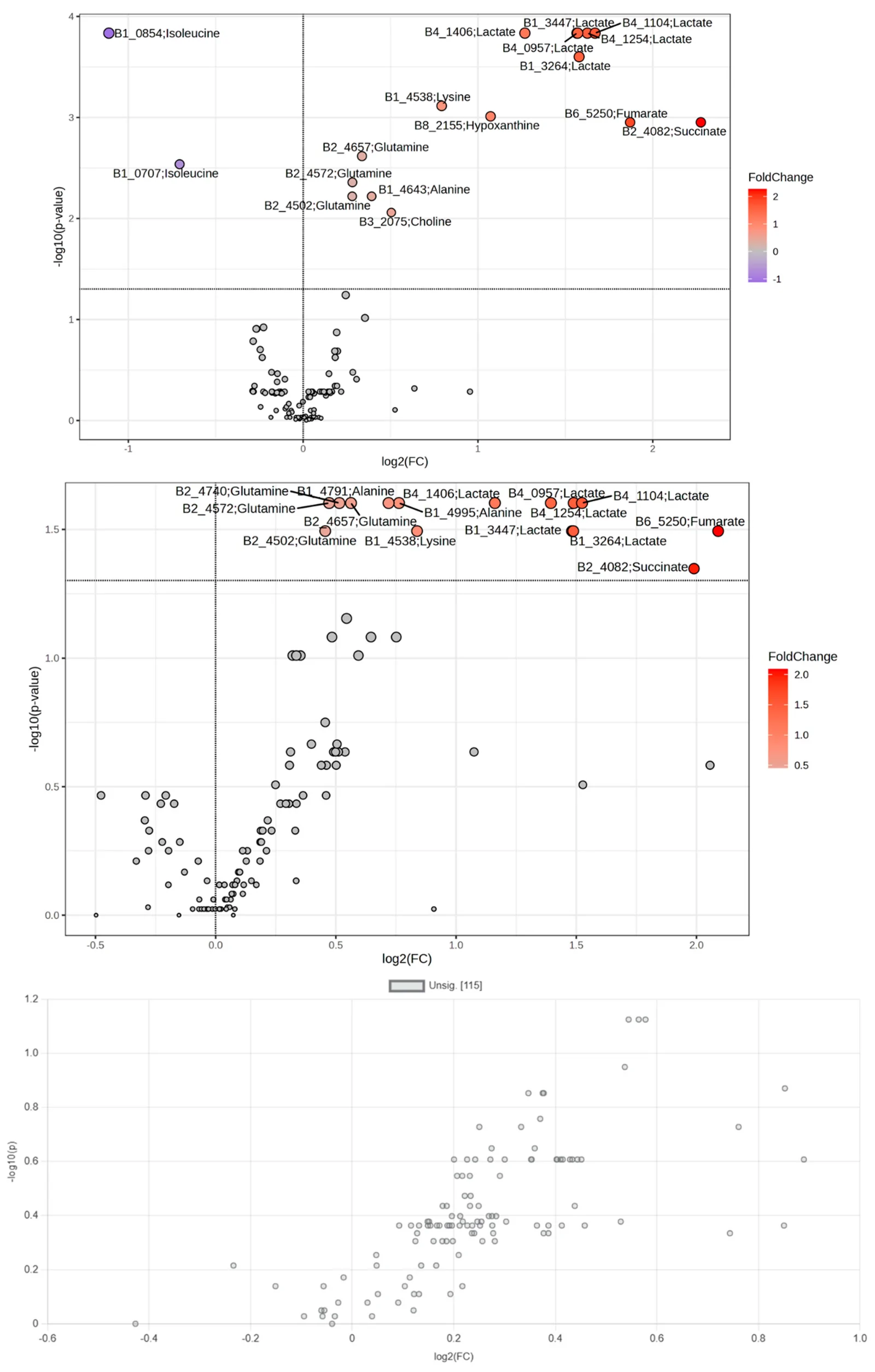
Volcano plots summarizing the univariate metabolite analysis across key experimental conditions. **Top**: “Asphyxia” vs. “Baseline”—significantly upregulated metabolites include lactate, succinate, fumarate, lysine, and hypoxanthine, while isoleucine was significantly downregulated. **Middle**: “Resuscitation” vs. “Baseline”—several metabolites, including lactate and glutamine, remain elevated post-resuscitation. **Bottom**: “Resuscitation” vs. “Asphyxia”—minimal statistically significant differences observed, indicating a metabolic shift toward recovery. Dotted lines represent the significance threshold (*p*  <  0.05) and log_2_FC cutoffs.

In the “Asphyxia” vs. “Baseline” comparison (Figure 11, top panel), a substantial number of metabolites demonstrated significant changes. Metabolites including lactate, succinate, fumarate, lysine, and hypoxanthine were markedly elevated in the “asphyxia” group, consistent with metabolic hallmarks of hypoxic stress. These metabolites exhibited log_2_ fold changes >1.5 and *p*  <  0.05, highlighting mitochondrial dysfunction and increased anaerobic metabolism. Conversely, isoleucine was significantly reduced in the “asphyxia” group, suggesting altered branched-chain amino acid metabolism.

The “Resuscitation” vs. “Baseline” comparison (Figure 11, middle panel) revealed persistent alterations post-ROSC (return of spontaneous circulation). Elevated levels of lactate, succinate, fumarate, and glutamine remained significantly different from “baseline”, suggesting incomplete metabolic recovery. The persistence of glutamine and alanine elevation further supports ongoing tissue repair or compensatory metabolic activity.

In contrast, the “Resuscitation” vs. “Asphyxia” comparison (Figure 11, bottom panel) demonstrated a clear reduction in the number and magnitude of statistically significant metabolites. Most of the features fell below the significance threshold, reflecting a trend towards normalization of the metabolome during early recovery. However, no metabolites reached significance at FDR-adjusted thresholds, reinforcing the intermediate biochemical status of the resuscitated animals.

Lactate, succinic acid and fumarate, which were found to be significantly elevated both at the timepoint of “asphyxia” and following resuscitation and ROSC, are implicated in the energy pathways and reflect the disturbance of the aerobic metabolism and the Krebs cycle following a severe hypoxic event. Moreover, some of the amino acids and metabolites that were found to be elevated following asphyxia may also reflect the disruption of the mitochondrial functions, the respective metabolic pathways, and the catabolic state that the organism adopts as an attempt to preserve and best utilize energy supply in conditions of oxygen deprivation.

Overall, these findings emphasize the distinct and dynamic metabolic shifts induced by asphyxia and partially reversed upon resuscitation. The univariate profile supports the multivariate OPLS-DA findings, particularly highlighting lactate, succinate, and fumarate as robust biomarkers of hypoxic insult.

Given the repeated-measures design of the study, univariate comparisons were performed using paired Wilcoxon signed-rank tests, matching samples from the same animal across timepoints. This approach was applied consistently across all pairwise comparisons (“Asphyxia” vs. “Baseline”, “Baseline” vs. “Resuscitation”, and “Asphyxia” vs. “Resuscitation”) and is reflected in the volcano plot analyses reported above (Figure 11).

Metabolites discussed in this section reflect nominal significance (raw *p*-value and log_2_FC threshold); FDR-adjusted results for the key discriminant metabolites are reported separately in Section 3.9.

### 3.7. Pathway Analysis

To assess the biological convergence of the identified metabolite signatures, pathway analysis was performed separately for each discriminant comparison using the Sus scrofa-specific KEGG library.

**Figure 12:**
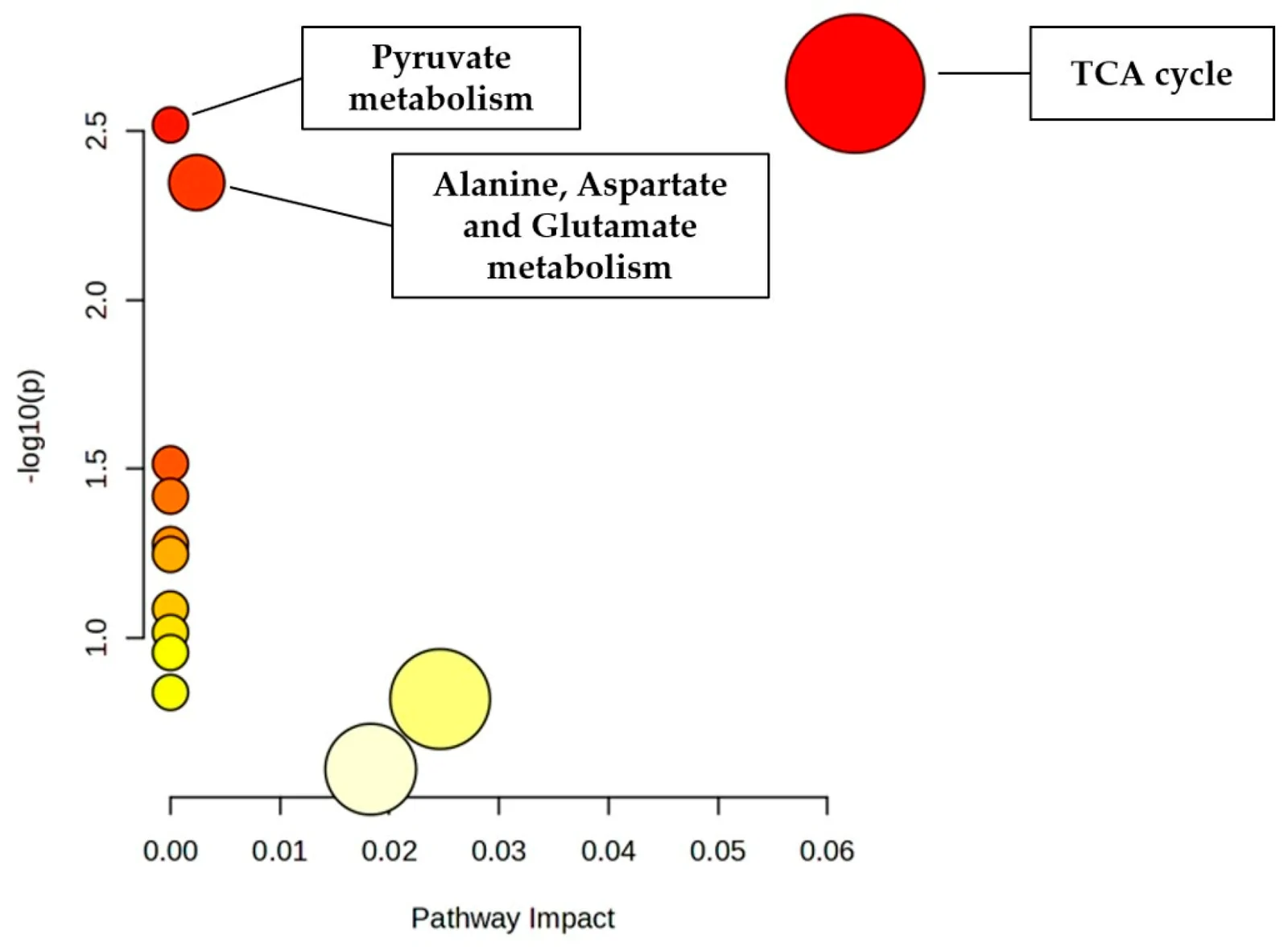
Pathway analysis bubble plot for the “Asphyxia”-vs.-“Baseline” metabolite signature (lactate, succinate, lysine, fumarate, hypoxanthine, isoleucine). Node color reflects statistical significance (−log10 *p*, darker red = more significant) and node size reflects pathway impact score. Sus scrofa-specific KEGG library, MetaboAnalyst 6.0. TCA cycle: Tricarboxylic Acid cycle.

For the “Asphyxia”-vs.-“Baseline” signature (lactate, succinate, lysine, fumarate, hypoxanthine, isoleucine), the citrate cycle (TCA cycle), pyruvate metabolism, and alanine, aspartate and glutamate metabolism pathways showed the strongest over-representation (raw *p* < 0.005 for all three; Figure 12). None of these reached significance after FDR correction (FDR ≥ 0.12), which reflects the limited statistical power of pathway enrichment testing when applied to a small, targeted metabolite set rather than an untargeted panel, and should be interpreted as descriptive support for pathway convergence rather than formal statistical confirmation.

**Figure 13:**
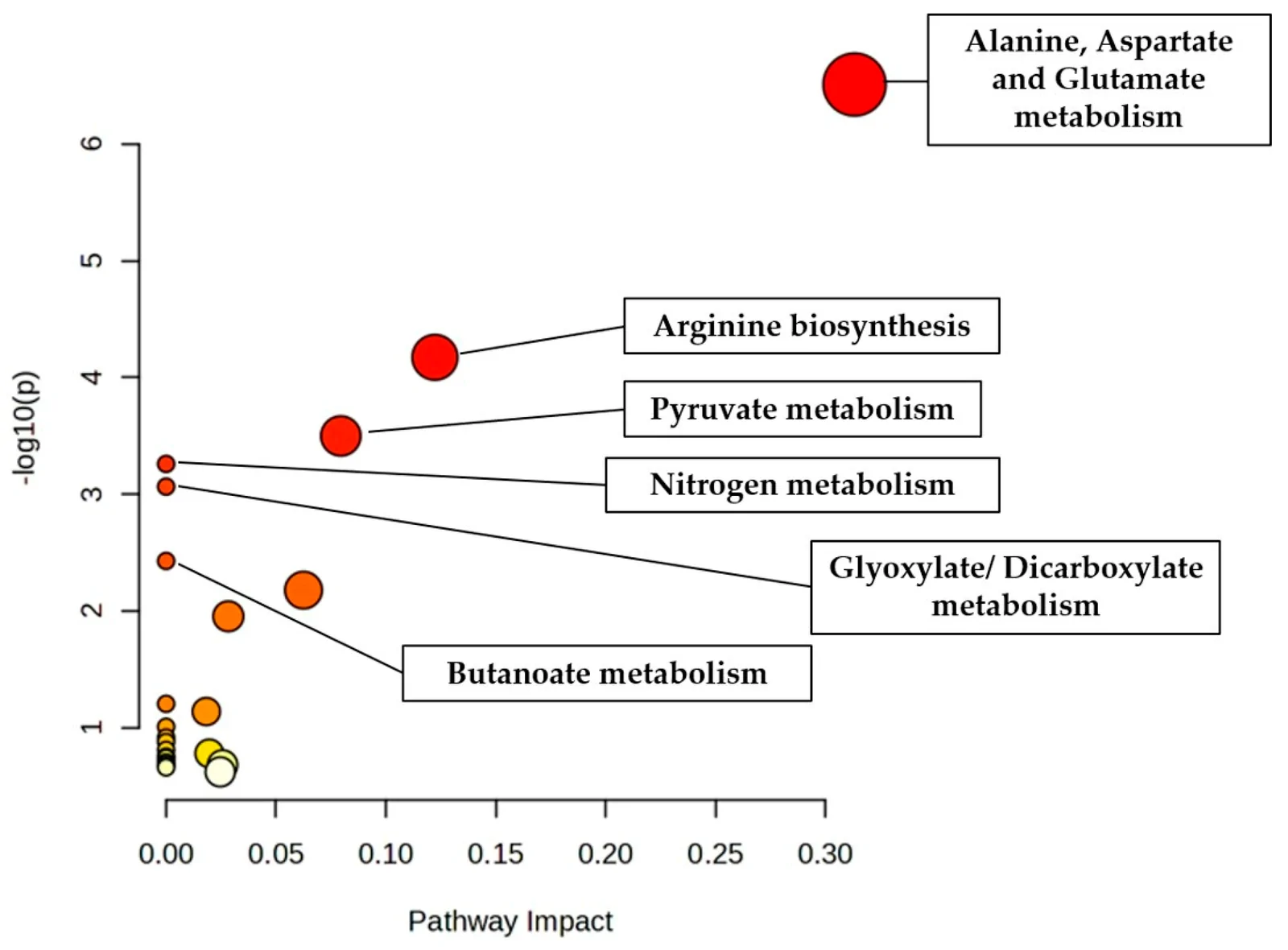
Pathway analysis bubble plot for the “Baseline”-vs.-“Resuscitation” metabolite signature (lactate, lysine, fumarate, hypoxanthine, succinate, acetate, alanine, glutamine, glutamate, choline). Node color reflects statistical significance (−log10 *p*) and node size reflects pathway impact score. Six pathways reached FDR < 0.05: alanine/aspartate/glutamate metabolism, arginine biosynthesis, pyruvate metabolism, nitrogen metabolism, glyoxylate/dicarboxylate metabolism, and butanoate metabolism. FDR: False Discovery Rate.

For the “Baseline”-vs.-“Resuscitation” signature (lactate, lysine, fumarate, hypoxanthine, succinate, acetate, alanine, glutamine, glutamate, choline), pathway analysis identified significant enrichment surviving FDR correction in six pathways (Figure 13): alanine, aspartate and glutamate metabolism (FDR = 2.5 × 10^−5^, impact = 0.31), Arginine biosynthesis (FDR = 0.0027), pyruvate metabolism (FDR = 0.0085), nitrogen metabolism (FDR = 0.011), glyoxylate and dicarboxylate metabolism (FDR = 0.014), and butanoate metabolism (FDR = 0.050).

The convergence of both signatures on central carbon/energy metabolism (TCA cycle, pyruvate metabolism) and amino-acid/nitrogen-handling pathways (alanine, aspartate and glutamate metabolism) supports the biological coherence of the persistent post-resuscitation metabolic signature discussed in Section 4.

### 3.8. Retrospective Power Analysis

Retrospective power analysis was performed for the three metabolites most consistently identified across comparisons (lactate, succinate, hypoxanthine), using Cohen’s *d* computed from the observed group means and standard deviations at the actual sample sizes used in this study (Table 5). Achieved power exceeded 0.99 for all three metabolites in both comparisons, reflecting the very large effect sizes observed (Cohen’s *d* ranging from 1.44 to 2.93) and indicating that this study was adequately powered to detect the reported differences despite the modest, pilot-study sample sizes.

### 3.9. Descriptive Statistics

**Figure 14:**
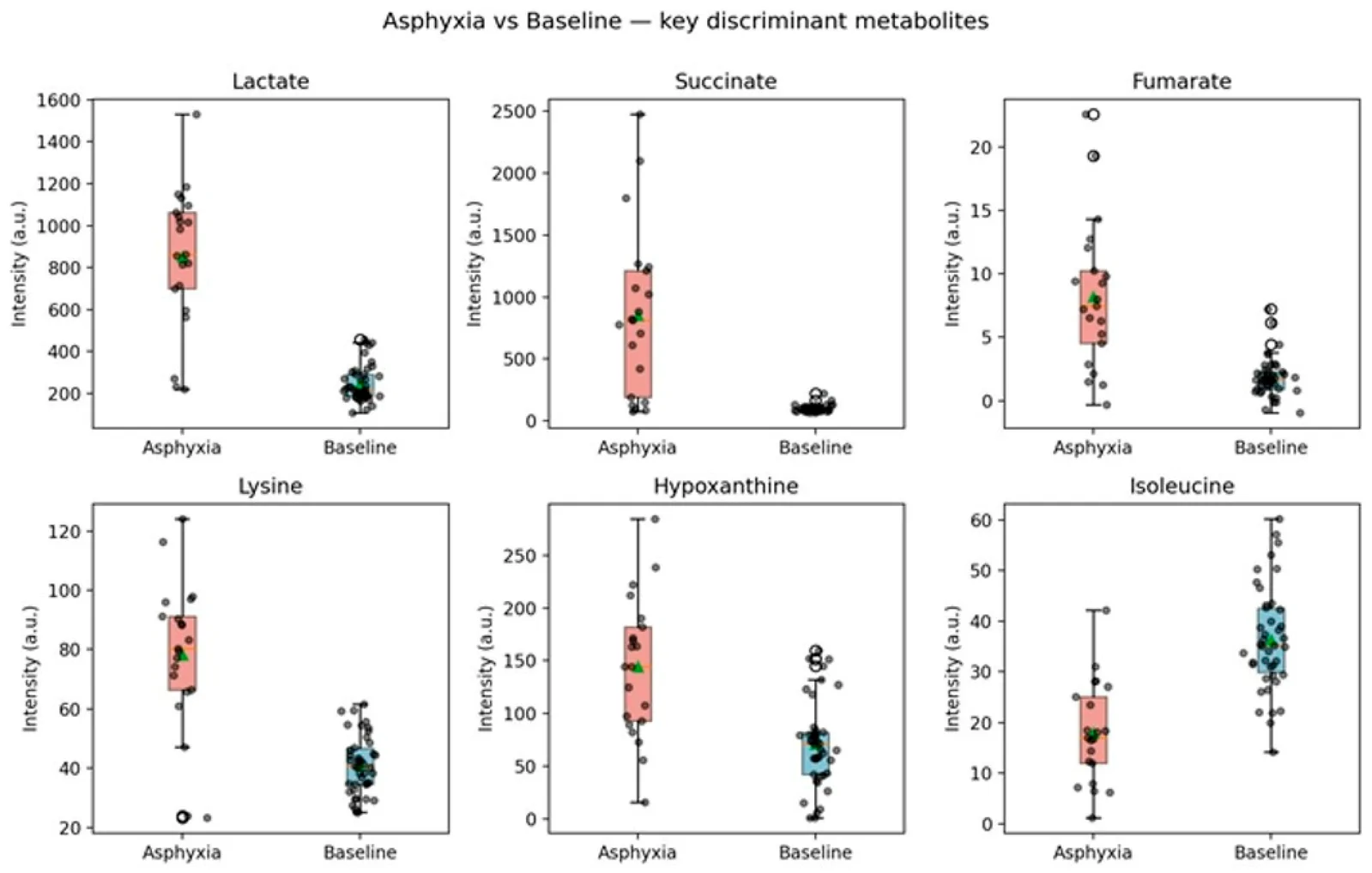
Boxplots of key discriminant metabolites, “Asphyxia” (red) vs. “Baseline” (blue). Boxes show median and IQR; triangles indicate group means. IQR: Interquartile Range, a.u.: arbitrary units.

**Figure 15:**
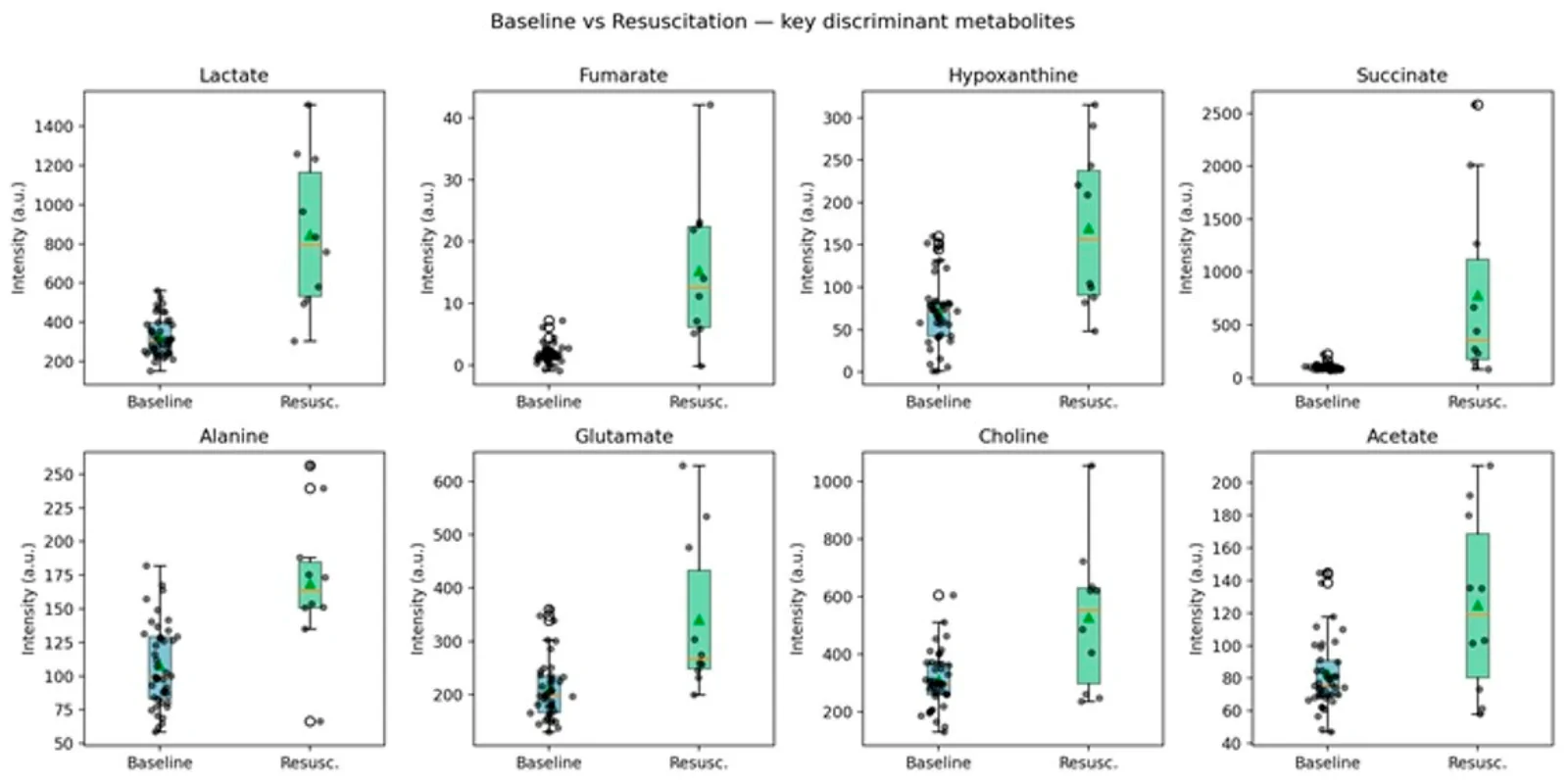
Boxplots of key discriminant metabolites, “Baseline” (blue) vs. “Resuscitation” (green). Boxes show median and IQR; triangles indicate group means. IQR: Interquartile Range, a.u.: arbitrary units.

Descriptive statistics (median and Interquartile Range) for the key discriminant metabolites in each comparison are presented in Table 6 and Table 7, alongside paired Wilcoxon signed-rank *p*-values and FDR-adjusted *q*-values, and the respective box plots are presented in Figure 14 and Figure 15. All metabolites previously identified as significant discriminants (Section 3.6) remained significant after FDR correction (*q* < 0.05), with the exception of alanine/aspartate and glutamate pathway markers already discussed.

Finally, the comprehensive results regarding the key discriminant metabolites in each comparison (descriptive statistics, paired Wilcoxon signed-rank *p*-values, FDR-adjusted *q*-values, AUROC/CI and VIP scores) are presented in Table 8 and Table 9.

## 4. Discussion

Over recent years, several studies have focused on correlating pediatric and neonatal conditions, such as perinatal asphyxia, with alterations in the metabolome. These studies aim to both identify novel biomarkers and also investigate the pathophysiological basis of the respective conditions.

In the case of the current study that was conducted in piglets, it is worth pointing out that the serum metabolic profile of swine has been shown to bear a significant compositional resemblance to that of humans in an experimental study that attempted to elucidate the metabolic phenotypes of the pig serum, urine and liver and kidney tissues and compare them to the respective phenotypes in humans [29]. Furthermore, pigs have been used as surrogates in clinical trials referring to the pediatric population due to the similarities in physiology, organ development and response to disease [30]. Additionally, several studies have attempted to elucidate the piglet metabolome in different conditions, such as IUGR, and biological fluids, such as cerebrospinal fluid, aiming at enriching the available data pool that can be further extrapolated and applied to the pediatric population [31,32].

As far as perinatal asphyxia is concerned, there is ongoing research in the metabolomic profile and dynamic alterations that characterize it, and studies have been performed on different biological fluids, such as plasma, urine, saliva and cerebrospinal fluid (CSF), as well as tissue specimens. It is worth pointing out that blood metabolomic alterations provide us with more rapid insight into the effect of hypoxia on the patient, while urine metabolome modifications are considered to be more delayed and affected by the renal function and clearance characteristics of each substance [12].

For the current study, we selected ^1^H NMR spectroscopy as the primary analytical platform because the aim was to obtain robust, reproducible and relatively high-throughput profiling of serum metabolites expected to change significantly after a severe hypoxic insult. Furthermore, the fact that NMR spectroscopy allows for the identification of novel chemicals in cases where no prior information is available and the discovery of unexpected biomarkers is one of the reasons why it was considered optimal for the design of the current study and chosen over alternative methods such as Mass Spectrometry (MS) [18]. Additional advantages of NMR spectrometry in comparison with Liquid Chromatography-Mass Spectrometry (LC—MS) and Gas Chromatography–Mass Spectrometry (GC—MS) that further supported its utilization in the current study, despite its lower analytical sensitivity, include its excellent reproducibility, minimal sample preparation, and non-destructive, non-selective, broadly quantitative profiling capabilities for abundant biofluid metabolites [20,33,34]. As far as the limitations of NMR are concerned, the most significant ones are the lower sensitivity (lower limit of detection ≥ 1 μmol/L) and need for larger sample volumes compared to MS [20]. However, given the design and context of the study, these limitations were not considered as restrictive, taking into consideration that the necessary sample volumes could easily be obtained through the central vascular catheters, and most of the metabolites of interest were expected to be above the detection threshold and not require derivatization. Moreover, several studies of different concepts and settings have been performed with the use of NMR spectroscopy on piglet serum, and it is therefore considered an appropriate and useful technique [34,35]. We therefore used NMR as a first-line discovery platform, while acknowledging that complementary MS-based analyses may be useful in future work to extend metabolite coverage.

### 4.1. Key Altered Metabolites

The present pilot study demonstrates that severe asphyxia in neonatal piglets induces a distinct serum metabolomic phenotype that is clearly separated from “baseline” and is incompletely restored 30 min after resuscitation and ROSC. The principal discriminant metabolites between the “baseline”-control state and “asphyxia” in the current study were lactate, succinic acid, fumarate, lysine, hypoxanthine and isoleucine. These findings are overall in accordance with the available literature. Furthermore, despite the lack of statistically significant differences between the “asphyxia” and “resuscitation” states, additional metabolites that were found to be elevated between “baseline” and “post-resuscitation” were acetate, alanine, glutamine, glutamate and choline. This pattern may reflect small cumulative changes that did not reach significance in the direct “Asphyxia” versus “Resuscitation” comparison but became evident when post-resuscitation samples were compared with “baseline”. Moreover, no difference was noted between “baseline” and “post-resuscitation” with regard to isoleucine, suggesting partial early reversal of this specific alteration.

It is also worth noting that, according to the results of the current study, lactate, succinate and hypoxanthine levels appear to be significantly correlated with the duration of the hypoxic insult. Although this observation needs to be confirmed with further studies on larger populations, it can prove very useful should it be applied in clinical settings. Being able to estimate the exact duration of asphyxia a newborn infant has undergone could guide the diagnostic and treatment modalities, thus greatly facilitating the overall management of our patients. On the other hand, taking into consideration the fact that the piglets that were exposed to asphyxia for a longer period of time were actually the ones that manifested with hemodynamic compromise later compared to their counterparts, a question arises as to whether certain compounds may actually reflect the activation of compensatory mechanisms and have a beneficial effect on the overall maintenance of homeostasis until the hypoxic insult is resolved.

Taken together, our findings support a model in which asphyxia induces a rapid serum metabolomic shift dominated by impaired oxidative phosphorylation, TCA-cycle (Tricarboxylic Acid cycle) disequilibrium and enhanced anaerobic metabolism. The persistence of several abnormalities within 30 min after ROSC suggests that early hemodynamic recovery does not equate to immediate metabolic restoration. Thus, the main finding of the study is not only the detection of an acute hypoxic metabolic signature but also the persistence of early post-resuscitation abnormalities despite hemodynamic recovery. The latter supports the candidacy of specific metabolites as potential markers of very early post-insult metabolic disturbance.

### 4.2. Comparison with Other Models

In an experimental model of perinatal asphyxia that was run by Beckstrom et al. [36] on non-human primates in an attempt to unveil novel perinatal asphyxia biomarkers, using two-dimensional gas chromatography coupled to time-of-flight mass spectrometry (GC × GC-TOFMS), ten metabolites were found to be significantly elevated in the animals that had been exposed to asphyxia compared to the control animals. Among these, succinic acid was highlighted as the most sensitive metabolite to asphyxia while lactic acid and leucine were also found to be markedly elevated in the asphyxiated animals, suggesting their potential as biomarkers of the condition.

Furthermore, Skappak et al. [37] designed and conducted a model of neonatal hypoxia–reoxygenation on male neonate piglets and obtained urine samples at critical times, which were processed with NMR spectroscopy, aiming at investigating the metabolomic profile in urine following hypoxia and the potential alterations between the animals that had been exposed to hypoxic conditions and the ones that served as controls. Among other metabolites, alanine, fumarate and lactate were found to be significantly elevated in the group of animals that had been exposed to hypoxia (FiO_2_: 0.10–0.13) for 2 h and further resuscitated with FiO_2_:1 for 30 min. The samples that were used for the comparison between the groups were obtained 3.5 h following reoxygenation. Although in this study hypoxia was implemented in a less acute and intense form and with longer duration compared to our study, and the overall observation time before the sample acquirement was longer, it can be anticipated that since hypoxia is a common endpoint of both experimental procedures, the metabolic pathways that are activated could be expected to bear resemblance to some degree. Moreover, the fact that in the study by Skappak et al. the analysis was performed on urine rather than blood, as was the case in the current study, can be considered as partly accountable for the differences in the rest of the metabolites that were highlighted in each study.

With regard to the effect of the duration of asphyxia and regimens of resuscitation on the plasma metabolome, a series of experimental studies have been performed. More specifically, in a study performed by Solberg et al. [38] on newborn piglets that were exposed to hypoxemia for different durations followed by resuscitation with either room air (FiO_2_: 0.21) or 100% oxygen mixture, the ratios of alanine to branched chained amino acids (Ala/BCAA) and glycine to branched chained amino acids (Gly/BCAA) appeared to be significantly correlated to the duration of hypoxia, while traditionally used biomarkers such as lactate, pH and Base Excess did not appear to be affected by it. In the same study that utilized mass spectrometric analysis, lactate, alpha keto-glutarate, succinate and fumarate were found to be significantly increased following hypoxia, but their concentrations appeared to decline following resuscitation (approximately 45 min post-termination of the resuscitation period). Additionally, with the exception of lactate, the levels of the rest of the metabolites appeared to decrease at different rates depending on whether hyperoxia (100% oxygen) or room air was offered during the resuscitation and stabilization. The decline was found to be faster in the room air group, thus indicating an earlier recovery of mitochondrial function according to the authors. In the case of the current study, the levels of the metabolites did not appear to be restored following resuscitation, and this can be partly attributed to the short period of observation following ROSC. Additionally, the supply of oxygen was adjusted based on the oxygen saturation of the animals and was increased to 100% in the cases requiring chest compressions according to the NLS algorithm. Therefore, our protocol was not standardized as per the oxygen mixture, and subsequently, no conclusions can be extracted regarding the effect of oxygen administration on the metabolomic modifications in our study subjects. However, it is worth mentioning that in our study, population lactate levels appeared to be correlated with the duration of asphyxia.

In a similar study [39] that attempted to simulate perinatal asphyxia via the exposure of neonate piglets to 8% mixture of oxygen for approximately 60 min, the metabolite that appeared to be most significantly elevated in the hypoxic neonates was choline while, among others, hypoxanthine, glutamine, and carnitine-fatty acids were increased, and free carnitine was decreased in the asphyxiated animals compared to the controls. This study utilized an untargeted liquid chromatography-time of flight mass spectrometry (LC-TOFMS) approach. Interestingly, in the blood samples that were obtained 120 min following resuscitation with room air, the metabolome was similar to the profile before commencement of hypoxia; therefore, the authors concluded that the metabolomics modifications can be considered to be transient. However, in an ensuing study [40] utilizing ultra-performance LC coupled to tandem MS (UPLC-MS/MS) and attempting to detect potential biomarkers of perinatal asphyxia whose concentration would change proportionally to the degree of injury during the course of the disease, choline, cytidine and uridine were significantly increased following exposure of the piglets to hypoxia, and choline levels remained elevated at a statistically significant degree even at 2 h following reoxygenation. However, at 9 h following reoxygenation, all metabolite concentration changes were found to be negligible. The authors concluded that the predictive value of lactate, which is currently considered as the gold standard in the assessment of perinatal asphyxia, can be further improved with the concurrent application of a panel of determined metabolites, and the levels of choline and its metabolites, either in the plasma or in the urine, are suggested to be potentially indicative of the duration of asphyxia when combined to lactate levels.

Furthermore, in an experimental study [41] using NMR spectroscopy and attempting to trace differences in the metabolic profile in plasma and urine of piglets that had been exposed to asphyxia and resuscitated with different protocols, lactate, succinate, fumarate, malate and alanine were elevated and subsequently decreased in the plasma of the animals that were asphyxiated, while choline was again found to increase with no subsequent decrease. However, no significant correlation was found between the plasma and urine metabolome modifications, and the authors concluded that plasma is superior to urine samples for real-time monitoring of acute conditions.

Conducting such studies in humans is difficult due to both ethical and practical constraints, as perinatal asphyxia is an acute condition that cannot always be anticipated prior to delivery. Therefore, prompt sample acquirement can prove difficult to perform. However, there are some available data in the literature, investigating the potential alterations in the metabolomic profile of human neonates mainly in urine samples but also, occasionally, in cord blood.

Reinke et al. [42] collected cord blood samples, which were processed with _1_H NMR spectroscopy, in an attempt to trace differential metabolic responses in asphyxiated neonates with or without manifestation of HIE. According to the results of their study, 18 metabolites were found to be present in different concentrations between the asphyxiated infants and their matched controls, while 13 metabolites (12 of which were overlapping with the previous set) were significantly altered between the infants presenting with HIE and their matched controls. More specifically, alanine, choline, creatine, glycerol, isoleucine, lactate, leucine, myo-inositol, pyruvate, phenylalanine, succinate, and valine were markedly elevated in both asphyxia and HIE infants, whereas acetone, betaine, creatinine, glucose, 3-hydroxybutyrate and O-phosphocholine were found to be elevated solely in the asphyxiated versus control group.

A study was run and presented by El-Farghali et al. [43] who compared cord blood metabolome obtained from neonates with perinatal asphyxia and HIE both between them and against samples from the control group. The blood samples were processed using ultra-performance liquid chromatography-mass spectrometry (UPLC-MS). Significant alterations in amino acids and acylcarnitines were highlighted in the cases compared to controls. Among others, the metabolic pathways of glycerophospholipids, arginine, proline, glutathione, purine, pyrimidine and thiamine appeared to be significantly affected. Therefore, the authors presented a clear discrimination between profiles of asphyxiated and non-asphyxiated infants as well as between cases with and without HIE.

Finally, a more recent study [44] utilized mass spectrometry-based metabolomic analysis on the cord blood of neonates at risk of developing HIE. The metabolites that were found to be most significantly altered in infants who experienced perinatal asphyxia or presented with HIE, compared to controls, were palmitoyl-L-carnitine, lactic acid, succinic acid and uridine. The authors attempted to correlate clinical data with metabolomic profiles in order to predict HIE in the most accurate way and differentiate between perinatal asphyxia and HIE.

### 4.3. Pathophysiological Pathways and Clinical Significance

Being a condition that is characterized by systemic oxygen and energy deprivation, perinatal asphyxia can be expected to induce a metabolic shift from oxidative phosphorylation towards anaerobic metabolism, aiming at providing the vital organs with the necessary energy to sustain life by utilizing the limited resources that are available. Therefore, as has already been shown in previous studies, several intermediates of anaerobic metabolism are expected to be elevated with a concomitant disruption of the Krebs cycle [45]. This is in accordance with some of the current study’s findings and can explain some of the alterations in the metabolic profile in the cases of asphyxia. Lactate, succinate and fumarate were elevated during asphyxia and remained abnormal following post-resuscitation stabilization, while several amino acid-related changes suggested activation of catabolic and compensatory pathways.

Lactate elevation is the most direct consequence of enhanced anaerobic glycolysis during oxygen deprivation [46]. The accumulation of lactate in the extracellular fluid contributes to metabolic acidosis, which compromises the overall homeostasis of the infant. However, it has also been suggested and verified in an in vivo study by Wyss et al. [47] that lactate can serve as an alternative cerebral energy substrate under hypoxic conditions, thus having a direct neuroprotective effect.

From a translational perspective, lactate is already one of the most clinically accessible metabolites because it can be measured through a quite accessible and timely performed laboratory test, or even through the use of most of the blood gas analyzers, in hospital settings. Therefore, it has long been used as a biomarker of tissue hypoxia, and there is a significant amount of data regarding its impact and clinical significance. With regard to perinatal asphyxia and HIE, lactate measurement has been considered to be the gold standard method for the clinical grading and decision making so far [48]. It has been suggested that a lactate concentration of >8 mmol/L, especially when associated with a base excess of >−12 mmol/L, is a reliable indicator of intrapartum asphyxia [49]. However, according to the available literature, the plasma lactate levels do not always correlate with the hypoxia duration and significance [50]. The diagnostic and prognostic value of lactic acid measurement is further enhanced, according to a recent study that has managed to associate the early lactate levels following perinatal asphyxia with the risk of developing oliguria in the first 24 h of life [51]. In addition to this, and apart from the already known predictive value of lactate for the severity of HIE and neurological morbidity over the first days of life, it has been recently shown to constitute an independent predictor of adverse outcomes in asphyxiated newborns who were treated with therapeutic hypothermia, thus supporting the prognostic relevance of lactate in the post-hypothermia era [52]. The persistence of lactate elevation after resuscitation and ROSC in the present study therefore supports the clinical relevance of our model and suggests that post-resuscitation sampling may still capture the metabolic imprint of the hypoxic insult.

Following restoration of the neonate’s circulation, lactate levels are expected to gradually decline and normalize. Nevertheless, according to some experimental studies and research protocols, persistently elevated lactic acid levels have been related to the degree of neonatal encephalopathy. This may reflect delayed clearance in view of hepatic and renal dysfunction, ongoing production owing to compulsive activity or tissue injury, or incomplete metabolic recovery [50].

Succinate provides a particularly informative mechanistic link between hypoxia, mitochondrial dysfunction and reperfusion injury. Succinic acid is markedly elevated in several experimental models of perinatal asphyxia, while in some cases it exhibits one of the most pronounced increases among all measured metabolites [36].

As a Krebs cycle intermediate, succinate is expected to accumulate following asphyxia. Its accumulation has been described as a “universal metabolic signature of ischemia”, and it is partly attributed to the reverse operation of succinate dehydrogenase, the reversal of the malate/aspartate shuttle, and fumarate overflow from purine nucleotide breakdown [53]. During oxygen deprivation and oxidative stress, mitochondrial NADH metabolism and TCA cycle flux are disrupted, while hydrogen peroxide has been shown to inhibit enzymes such as aconitase, α-ketoglutarate dehydrogenase and, to a lesser degree, succinate dehydrogenase [36,54]. Upon reperfusion, accumulated succinate can be rapidly oxidized, driving reverse electron transport and reactive oxygen species (ROS) generation [53].

Succinate is not merely a passive marker of injury. Through succinate-coenzyme Q reductase it links the TCA cycle to the respiratory chain, and it can also function as an intracellular and extracellular signaling molecule involved in hypoxic adaptation, inflammation, gene regulation and epigenetic modulation [55,56,57,58,59,60]. Elevated succinate may therefore reflect both the severity of mitochondrial injury and the activation of adaptive responses to oxygen deprivation.

Regarding its clinical significance, elevated succinate in severe HIE has been linked to hypoxia-inducible factor-1alpha (HIF-1alpha) signaling. Succinate, hypoxia and reactive oxygen species can inhibit prolyl hydroxylase activity, thereby promoting HIF-1alpha stabilization and accumulation. HIF-1alpha has been implicated in neurotoxicity via several mechanisms, including blood–brain barrier disruption, edema, inflammation and necrosis [42,61]. Interestingly, HIF-1α inhibition has been found to attenuate hypoxic–ischemic brain injury in neonatal rats, further supporting this pathological role [62]. Succinate has also been included in a cord-blood metabolite index for HIE risk, later associated with a 3-year neurodevelopmental outcome [42,63]. Furthermore, lactate correlated with succinate in an experimental study investigating the metabolome and potential biomarkers in cord blood samples in an attempt to differentiate between perinatal asphyxia, HIE and controls [44].

Overall, succinate oxidation during early oxygen supply disruption has been characterized as an evolutionary, protective and adaptive mechanism of tissue energy homeostasis. Interestingly, succinate-containing compounds have been tested in several acute settings and have been found to have energotropic and antihypoxic effects, thus acting as protective factors in cases of severe hypoxia [55].

According to the findings of the current study, it can be concluded that both lactate and succinate remain persistently elevated not only at the time of asphyxia but also 30 min following resuscitation and ROSC. Their concomitant persistence suggests ongoing mitochondrial dysfunction and incomplete recovery of aerobic metabolism during the early post-resuscitation period. Because both metabolites correlated with the duration of the hypoxic insult, they may potentially serve in the development of a combined index reflecting the duration of perinatal asphyxia. While these metabolites may be useful as biomarkers of injury severity, further studies correlating their levels with HIE grade, brain injury and long-term neurodevelopmental outcome are required before definitive prognostic claims can be made.

Being an intermediate in the Krebs cycle, fumarate is also expected to increase following exposure of the animals to asphyxia, as observed in the present study. Fumaric acid is produced through oxidation of succinate by the enzyme succinate dehydrogenase and further converted to malate by the enzyme fumarase. However, under hypoxic conditions and subsequent inhibition of O2 reduction, fumarate can act as an electron acceptor and be reduced to succinate through reverse succinate dehydrogenase activity, further contributing to the accumulation of succinate but also enabling NADH reoxidation [12,64]. The restoration of fumarate levels following resuscitation has been shown to occur faster when resuscitation is offered with FiO_2_: 21% rather than 100%, and this was the case with succinate and alpha-ketoglutarate as well, in an experimental study of hypoxemia and reoxygenation in newborn piglets [38]. This observation was considered by the authors as indicative of a delayed restoration of the cellular metabolism when excessive oxygen is used.

The therapeutic literature on fumarate derivatives is biologically interesting. Dimethyl fumarate, a fumarate derivative used in other clinical contexts, has shown antioxidant and cell-protective effects in experimental ischemia–reperfusion injury [65]. In neonatal rat models of severe hypoxic–ischemic brain injury, dimethyl fumarate has been shown to have a remarkable neuroprotective effect [66], while in a pediatric porcine model of asphyxia-induced in-hospital cardiac arrest, it appeared to exert favorable and therapeutic effects on mitochondrial structure and function in several tissues including the brain and myocardium [67]. These findings suggest that fumarate-related pathways may be relevant to mitochondrial stress responses. However, the present study did not test dimethyl fumarate or any therapeutic intervention targeting fumarate metabolism. Therefore, serum fumarate should be interpreted here primarily as a candidate marker of TCA-cycle perturbation and incomplete early mitochondrial recovery.

Lysine is an essential amino acid that is particularly abundant in neuronal proteins. Given its significant contribution to epigenetic alterations through methylation and acetylation, it can be expected that lysine plays a very important modulatory role in both physiological cellular processes and intercellular interaction [68]. Since mammals cannot synthesize lysine de novo, the elevated levels observed following asphyxia in the case of our study are more plausibly related to augmented protein catabolism and breakdown, impaired mitochondrial lysine degradation, or both. The lysine degradation pathway (reflected by the levels of lysine derivatives in the urine metabolome) has been reported to be disrupted in urinary metabolomics from asphyxiated neonates treated with therapeutic hypothermia, where lysine degradation products differed from controls at birth and changed during treatment, suggesting gradual restoration of the pathway [69].

In a recent study [70] applying metabolomics in serial urine samples obtained by newborns that had undergone perinatal asphyxia and were managed with therapeutic hypothermia during the course of the hypothermia treatment, L-lysine was among the four metabolites that were found to be discriminatory between the groups that did or did not manifest with HIE based on the brain MRI results. Therefore, L-lysine is indicated by the authors as a potential biomarker that could assist in the prediction of the neonates that develop HIE following perinatal asphyxia. Additional experimental data suggest that lysine administration following ischemic incidents, alone or in combination with arginine, may reduce edema, infarct size or glutamate-mediated neuronal activity [71].

Hypoxanthine, a metabolite deriving from the degradation of purines, has long been described to elevate in hypoxia, including fetal and neonatal asphyxia [72]. The fact that it has been reported to increase in a time-related manner depending on the duration of the applied asphyxia in experimental models further supports its eligibility as a potential biomarker, reflecting the duration and time of onset of perinatal asphyxia [73,74]. This is consistent with the findings of the current study, which indicate a statistically significant positive correlation between hypoxanthine concentration and asphyxia duration. The depletion of adenosine triphosphate (ATP) as an energy source, characterizing hypoxic conditions, enhances adenosine monophosphate (AMP) degradation to preserve energy levels. Subsequently, purine nucleotides and nucleosides are formed and further metabolized to hypoxanthine. Under energy-depleted conditions, the inhibition of both salvage and degradation pathways results in hypoxanthine accumulation [75]. Upon reoxygenation, the degradation of hypoxanthine is restored. The study of hypoxanthine metabolism and its correlation with oxidative stress provided the impetus for the revision of neonatal resuscitation protocols and the thorough study of the negative impacts of hyperoxia on neonatal survival and metabolic recovery [76]. Subsequent studies supported the above assumption, and the superiority of resuscitation with room air rather than 100% oxygen has been confirmed in patient series [77]. Furthermore, elucidating these pathways and their relation to HIE has driven trials evaluating allopurinol, a xanthine oxidoreductase (XOR) inhibitor, as an adjuvant neuroprotective therapy in the management of neonates with perinatal asphyxia [75].

In the current study, isoleucine levels decreased following asphyxia compared to the “baseline” sampling, a change that was not preserved following resuscitation. This finding contrasts with most of the available literature so far, since the majority of blood metabolomics studies have reported increased isoleucine in perinatal asphyxia or HIE [42,50,78]. However, reoxygenation following hypoxia has been described to lead to a decrease in blood isoleucine compared to the samples obtained after asphyxia [12]. Hence, further investigation is required in order to clarify the exact dynamics and role of isoleucine in asphyxia. Isoleucine is an essential branched-chain amino acid (BCAA). The first step of the degradation of BCAA during periods of fasting takes place in skeletal muscle and leads to the generation of branched-chain keto acids as well as glutamine and alanine [79]. The subsequent metabolism of these products leads to the production of ketogenic and gluconeogenic substrates that can enter the TCA cycle or be processed in different ways. Therefore, the suppressed isoleucine levels observed after asphyxia and the simultaneous elevation of both alanine and glutamine could reflect accelerated isoleucine catabolism as an adaptive response to hypoxia. Similarly, BCAAs appear to be metabolized during physical exercise for energy supply, and that is believed to prevent protein degeneration and muscle enzyme release while promoting the recovery of muscle function [80]. The catabolism of isoleucine during hypoxia may represent an aspect of the same mechanisms’ activation. A decrease in isoleucine has also been described in adult humans exposed to high-altitude hypoxia [81]. Although this refers to an entirely different setting compared to our study, it may reflect shared pathophysiological pathways involved in the systemic response to hypoxia. The decrease in isoleucine following asphyxia that was observed in the current study contrasts with reports of increased branched-chain amino acids in other models and in human cord-blood metabolomic studies of perinatal asphyxia and HIE [42]. This discrepancy could be attributed to several factors, such as differences in species, analytical platform, severity and duration of the hypoxic insult, and especially the timing of sample collection. According to the present study protocol, the “asphyxia” samples were obtained at the time of hemodynamic compromise, thus reflecting a very early phase of severe hypoxic stress. During this state, reduced circulating isoleucine may indicate increased tissue uptake and utilization of branched-chain amino acids as alternative substrates through transamination and entry of their carbon skeletons into mitochondrial energy pathways. By contrast, increased isoleucine or other branched-chain amino acids reported in later samples or in cord blood may reflect delayed proteolysis, cellular injury or impaired clearance after a longer hypoxic–ischemic interval. The absence of a persistent isoleucine difference between “baseline” and “post-resuscitation” samples in our study may further suggest that this alteration is transient and partly reversible during the early post-resuscitation period.

Furthermore, the fact that isoleucine is mainly metabolized in skeletal muscle rather than the liver may also account for the different response compared to the other metabolites that were characteristically found to be elevated following asphyxia. With regard to the potential impact of the above on long-term health and homeostasis, it is worth pointing out that beyond muscle recovery, isoleucine and the other BCAAs are involved in key cellular signaling pathways [81,82]. Therefore, it will be very interesting to elucidate the potential implications of the isoleucine decrease for the overall phenotypical expression and homeostasis of the involved organisms in future studies.

As previously mentioned, several metabolites were significantly elevated in the blood samples retrieved 30 min after resuscitation and ROSC compared with “baseline”, namely acetate, alanine, glutamate, glutamine and choline. One possible explanation is that resuscitation and reoxygenation themselves affected the involved pathways, leading to the accumulation of these substrates or products. However, given the absence of statistically significant differences between the “asphyxia” and “resuscitation” samples, it may be more reasonable to speculate that the relevant alterations were progressive and that the activated pathways were not inhibited or reversed within 30 min post-ROSC.

Alanine is a non-essential amino acid synthetized from pyruvic acid, and it constitutes an intermediate acid in several metabolic pathways that are involved with energy management. More specifically, it plays a pivotal role in gluconeogenesis; therefore, it is reasonably expected to be significantly elevated under anaerobic circumstances. Alanine can also be converted to pyruvate that can be further processed for energy supply through the TCA cycle [44]. Alanine has been found to be consistently elevated in blood, cerebrospinal fluid (CSF) and urine samples of neonates that have been exposed to perinatal asphyxia, with or without the manifestation of HIE [12,70,83]. Furthermore, as previously mentioned, the ratio alanine/branched-chained amino acids, together with the ratio glycine/branched chain amino acids, have been found to be indicative of the duration of hypoxia in an experimental model of neonatal hypoxia in neonate piglets [38]. It can be concluded that it can serve as a promising biomarker in the management of perinatal asphyxia.

Choline is metabolically linked to lipid- and folate-dependent one-carbon metabolism, and it has been reported to be involved in several diseases’ pathogenesis [84]. Some of its roles in mammalian physiology and homeostasis include the formation of the neurotransmitter acetyl-choline in the neuronal tissues, the synthesis of phospholipids that are involved in the composition of the mammalian cellular membranes, the assembly and secretion from the liver of very low-density lipoproteins (VLDL), and the formation of methionine from homocysteine. Interestingly, through the latter, choline and betaine can affect gene transcription and genomic imprinting [84]. Its physiological breadth makes interpretation complex. In the context of perinatal asphyxia, elevated choline may reflect membrane injury, altered phospholipid metabolism, mitochondrial dysfunction or blood–brain barrier disturbance rather than a single pathway disturbance.

Choline blood levels have been reported to increase after perinatal asphyxia and in HIE in several studies [12,40]. In piglet hypoxia–reoxygenation models, choline can follow a pattern similar to lactate and remain significantly elevated for at least 2 h after reoxygenation; combining choline-related metabolites with lactate has improved assessment of asphyxia duration in experimental settings [40]. Choline has also been incorporated with hypoxanthine and 6,8-dihydroxypurine into a metabolite score for perinatal asphyxia and has been associated with HIE severity in human cord-blood studies [48,85].

Choline is actively transferred from the mother to the fetus through the placenta, and eventually, the levels of choline in neonatal circulation are significantly higher compared to the mother. Furthermore, the developing neonatal brain has been found to have a high-capacity transporter for choline across the BBB, and according to experimental data, choline seems to have a significant effect in the neurodevelopment of the infant [84]. Finally, experimental models that involve the administration of medical nutrition rich in choline among other nutrients from birth onwards to neonate animals exposed to perinatal hypoxia have highlighted a beneficial effect on neuroinflammation and lesion size, particularly in male neonates, and a favorable cognitive outcome in both genders [86].

Glutamine is a non-essential amino acid that participates in several critical metabolic pathways. More specifically, it plays an important role in acid–base balance regulation through ammonia genesis, while, through deamidation, it leads to the formation of glutamate. The latter is a very significant substance for neurotransmission, since L-glutamate is a primary excitatory neurotransmitter of the central nervous system. Moreover, glutamate serves as a precursor for γ-aminobutyric acid (GABA), an inhibitory neurotransmitter. Τhrough conversion to α-ketoglutarate, glutamine interacts with the TCA cycle, and it can also constitute as a substrate for gluconeogenesis [87]. Induction of gluconeogenesis may mechanistically partly explain the elevation of plasma glutamine observed in several studies of perinatal asphyxia, including the current study [45,83]. Moreover, the cellular damage and the generalized catabolic state that occur in cases of oxygen deprivation may also contribute to this accumulation. Finally, experimental models of hypoxia in mice have shown that hypoxia acclimation leads to increased production of glutamine and glutamate from the astrocytes [88].

As already mentioned, glutamate is an excitatory neurotransmitter that has been found to be elevated following perinatal asphyxia in several studies and has also been monitored in cases of therapeutic hypothermia (TH) [12]. Interestingly, according to the available literature, glutamate elevation following asphyxia is a primary driver in the pathophysiology of HIE, since the glutamate released in response to hypoxia triggers widespread excitotoxicity, causing secondary cerebral damage [12,83]. Furthermore, TH’s capacity to reduce glutamate levels is considered as one of the factors contributing to its beneficial effect. It is worth mentioning that the accumulation of glutamate in the synaptic cleft contributes to glutamate receptor overactivation, and this is considered as a form of vulnerability of the developing brain to hypoxic damage that is mediated by the influx of calcium into the cells [89]. Consequently, monitoring and, ideally, attempting to reduce the accumulation of glutamate appears to be a crucial target in the overall management of perinatal asphyxia.

Overall, the metabolomic alterations observed after asphyxia and resuscitation support previous models of impaired oxidative phosphorylation, TCA cycle disruption, and mitochondrial dysfunction in hypoxic tissues. Energy metabolism therefore appears to be one of the main pathways affected by asphyxia and incompletely restored within 30 min after ROSC. An important observation was that no metabolite survived FDR correction in the “Asphyxia” versus “Resuscitation” comparison, whereas several metabolites remained different when post-resuscitation samples were compared with “baseline”. This pattern suggests that the 30 min post-ROSC state is metabolically intermediate: resuscitation initiates partial movement away from the asphyxia phenotype, but early metabolic recovery remains incomplete. This interpretation is also consistent with the PCA and OPLS-DA findings, where the “resuscitation” group shifted towards but did not overlap with the “baseline” cluster.

The combined lactate–succinate–fumarate–hypoxanthine pattern is particularly coherent because it links anaerobic glycolysis, Krebs-cycle disequilibrium, ATP depletion, purine degradation and reperfusion-associated oxidative stress. The accompanying amino acid- and choline-related changes further suggest catabolic remodeling, membrane perturbation and compensatory substrate use.

Due to their early elevation and relative persistence post-resuscitation, serum metabolites measured in the current study—particularly lactate, succinate, fumarate, choline and selected amino acid-related signals—appear promising as candidate components of a biomarker panel for early bedside diagnosis and risk stratification. In a clinical setting, a metabolite signature that remains detectable after birth or after initial resuscitation could help identify neonates who may benefit from closer monitoring, therapeutic hypothermia when indicated, or future adjuvant neuroprotective approaches. Such a panel could eventually be incorporated into rapid analytical workflows, targeted NMR-based protocols or point-of-care decision algorithms. However, this translational application requires external validation in human neonates, evaluation of turnaround time and analytical feasibility, and correlation with clinically meaningful outcomes prior to the systematic application and utilization in the clinical setting.

In view of the animal experimentation principle of *refinement*, the piglets received medication for anesthesia, analgesia and muscle relaxation over the course of the experiments as described in the experimental protocol. However, several anesthetic drugs have been shown to interfere in the mitochondrial metabolism and TCA cycle. As far as the medication administered during the initial sedation is concerned, ketamine in moderate doses has been found to lead to increased levels of lactate, while reducing branched-chain amino acids and tyrosine [90]. Atropine has been shown to alter the cellular metabolome, affecting certain amino acid biosynthesis pathways, the metabolism of cysteine and methionine, as well as the Krebs cycle [91], whereas the potential effects of midazolam on the metabolomic profile have not been elucidated. However, all of the animals received the same doses per kg via the same route; therefore, the initial sedation is not expected to significantly affect this study’s results. In a recent study investigating the acute effect of certain anesthetics at moderate doses on the human metabolome, propofol was documented to alter lipoproteins, fatty acids, glycerides and phospholipids while slightly increasing glycoprotein acetylation [90]. Propofol has also been shown to suppress carnitine metabolism in hepatocytes and reduce intracellular amino acid levels [92]. Furthermore, it has been suggested that propofol has a protective effect on ischemia/reperfusion-associated cell injury, which might be partly mediated by alterations in the citrate cycle and purine metabolism as well as the lipid metabolism [93]. While the potential effect of cis-atracurium on metabolism has not been uncovered, fentanyl overdose has been shown to interfere in the glucose–alanine cycle, Warburg effect, gluconeogenesis, lactose degradation, and glutamate metabolism at the liver, among other pathways [94]. Moreover, metabolomics studies have indicated that the intake of opioids results in an elevated energy demand, particularly in male subjects, reflected through the increase in medium and long-chain acylcarnitine levels, while additionally disrupting the pathways associated with catecholamines biosynthesis [95]. Synthetic fentanyl analogs, such as carfentanil, have also been shown to affect the linoleic acid, arachidonic acid and glutathione metabolism pathways [96]. With regard to fentanyl and cis-atracurium, the cumulative doses that the animals received in the current study were similar between the different experimental groups. As far as the potential effect of propofol on the metabolome and the results of our study is concerned, in the current study, no significant correlation was observed between the propofol dose and the levels of the metabolites of greatest mechanistic concern. It can therefore be concluded that propofol administration did not significantly affect the findings of our study.

### 4.4. Study Limitations and Future Perspectives

The fact that the current study was performed in a neonatal rather than a strictly perinatal model of asphyxia and resuscitation can be considered as a limitation. However, it would not be feasible to apply a model directly at birth while neonatal models allow for clinically relevant cardiorespiratory monitoring, invasive instrumentation, controlled induction of asphyxia, and serial biological sampling. Furthermore, the first 4 days of life are considered part of the early neonatal period, which closely resembles the transition at birth. Therefore, the findings can be considered as applicable to the events occurring at birth. In addition to this, a substantial amount of the available literature has also been obtained from similar models, and there is extensive experience in their performance. Furthermore, as already mentioned, swine resemble humans in several anatomical and physiological aspects [38,97] and are therefore frequently used in basic research. Consequently, although our model does not reproduce an intrapartum event at the exact time of delivery, it provides a well-established and translationally relevant experimental approximation of early perinatal asphyctic injury.

The number of samples is another limitation. A larger number of animals, especially in the “asphyxia–resuscitation” group, might have allowed the identification of metabolomic profiles associated with favorable outcome or with prolonged tolerance to asphyxia before hemodynamic compromise. It was fairly interesting that in the present study, the duration of asphyxia varied substantially, ranging from 1.25 to 37.25 min, suggesting that some animals may have been better conditioned to tolerate hypoxia before decompensating. If such patterns are elucidated in future studies, they could facilitate the management and treatment of this severe condition. However, consistent with the 3 Rs principle of laboratory animal experimentation (Replacement, Reduction, Refinement), no further experiments were performed [98].

Additionally, while bootstrap-derived 95% confidence intervals supported the discriminative performance of the reported biomarkers, these intervals were notably wider for the “baseline”-vs.-“resuscitation” comparison, reflecting the small size of the “resuscitation” group (*n* = 10); AUROC estimates for this comparison, particularly for choline and alanine, should therefore be interpreted with appropriate caution pending validation in larger cohorts. Although within-animal correlation was addressed using paired non-parametric testing, the modest sample size of this pilot study limited the feasibility of a full mixed-effects modeling framework; future studies with larger cohorts should consider hierarchical/mixed-effects models to more comprehensively account for the repeated-measures design.

The definition of asphyxia based on hemodynamic criteria has been applied in several experimental models investigating aspects of perinatal or neonatal asphyxia [23,99]. The relatively large variation in the duration of asphyxia before blood sampling may have affected sample homogeneity and is therefore a confounding factor. At the same time, it may reflect the variability encountered in clinical practice. More specifically, when medical staff are called to manage a newborn presenting with signs of asphyxia at birth, the exact onset of the hypoxic insult is usually unknown, and diagnosis depends substantially on clinical presentation. Therefore, the hemodynamic compromise, which was the timepoint of blood sampling defined as “asphyxia” in the current study, can be considered as reflective of real-life settings. Furthermore, it is worth mentioning that the response to hypoxia is also a sequence of pathophysiological alterations rather than an all-or-nothing phenomenon, and individual response and tolerance to hypoxia is likely affected by individual traits. Once the metabolomic fingerprint of perinatal asphyxia is better defined, future studies could potentially include serial blood sampling at defined timepoints during the timespan of asphyxia. This could allow for a reverse diagnostic procedure in which the exact metabolomic alterations in blood samples from neonates undergoing asphyxia may facilitate the estimation of the exact duration of asphyxia prior to delivery and therefore further guide therapeutic management. Moreover, it would be fairly interesting to investigate the correlation of the metabolomic profile of the animals subjected to asphyxia with the duration of asphyxia until hemodynamic compromise, as well as the duration and extend of resuscitation until ROSC. Should such a correlation be highlighted, it could provide us with valuable information regarding certain characteristics and metabolomic phenotypes that constitute a type of “benefit” with regard to response and tolerance to hypoxia. Nevertheless, correlating metabolic profiles with the duration and intensity of asphyxia and resuscitation would require larger sample sizes.

The absence of statistically significant differences between asphyxia and 30 min post-resuscitation might be considered predictable, since the homeostatic mechanisms are expected to require an amount of time to recover following such an intense insult. However, most comparable studies include sampling at 1–2 h after reoxygenation. Therefore, the finding that the metabolomic profile remains disturbed at 30 min post-ROSC is clinically relevant. It suggests that the candidate biomarkers that were identified in the present study may remain detectable when blood sampling immediately after the insult is not feasible. More specifically, the validity of the molecules that were suggested as potential biomarkers from the findings of our study can be speculated to remain unaltered over this time span. The 30 min post-resuscitation time interval was mainly selected in order to capture early metabolic alterations following resuscitation and reoxygenation. In clinical settings, samples are often collected after admission to the neonatal intensive care unit rather than at the exact time of asphyxia. Nevertheless, we acknowledge that certain recovery-related metabolic shifts are expected to occur later on, and future studies involving serial sampling at more frequent and extended intervals would be expected to better define the exact trajectory of recovery for each metabolite and pathway.

Additional experimental limitations include the absence of a sham-resuscitation or resuscitation-only control group and the potential influence of anesthesia, mechanical ventilation and invasive instrumentation on the metabolome. However, all animals underwent the same initial preparation under anesthesia, and control animals remained ventilated and monitored for a comparable period. This should have reduced, although not eliminated, procedure-related bias. According to the analysis of the current study’s data, the exposure of the animals to anesthetic medication is not expected to be a significant confounder. Oxygen administration during resuscitation was guided by oxygen saturation and neonatal resuscitation algorithms rather than standardized as an experimental oxygen-mixture intervention; therefore, the present study cannot assess oxygen-dose-specific metabolomic effects.

The absence of long-term follow-up is another central limitation. Because animals were humanely euthanized 30 min post-ROSC in the “resuscitation” group, the present study cannot determine whether the identified metabolites predict neurological outcome, organ dysfunction or survival. Accordingly, any prognostic implications should be considered hypothesis-generating and speculative. Future studies could include serial sampling over longer intervals, ideally extending to 24–72 h, and should incorporate neurological, histopathological and functional outcome assessments in order to validate candidate prognostic biomarkers.

The fact that the population of the current study consisted exclusively of female piglets was mainly dictated by the design of the initial study that was undertaken. Therefore, our results can be considered to refer to a specific part of the general population, and this could be speculated to be a limitation of the study. Several studies and clinical observations have indicated that certain outcomes affecting development appear to be gender-dependent. More specifically, HIE has been shown to have a less favorable outcome in males compared to female infants, whereas the mortality rate of male infants is higher than females. Experimental data obtained from a recent study in piglets highlighted not only that male subjects exposed to hypoxia–ischemia were more vulnerable than their female counterparts, but also that the brain injury that was caused presented with a region-specific response depending on sex, and the pattern of cell death differed between the two sexes [100]. Furthermore, the long-term consequences of hypoxia in males are more severe compared to females according to experimental research data [101,102,103]. Owing to these observations, the term “male disadvantage” has been introduced with regard to the perinatal period [104]. The exact mechanism that accounts for this dimorphism has not been elucidated yet, but hormone-related actions as well as different cell death patterns have been speculated to partly contribute. According to the available data, several metabolic features, such as amino acids and carnitines, that constitute the neonates’ metabolome may be gender-related, although there is no consensus between the different studies [105,106]. This variation in the metabolic profile could partly reflect the clinical differentiation in the infants’ response to hypoxia–ischemia based on the sex. Therefore, it can be assumed that the use of animals of the same gender in the current study eliminates a potential confounding factor and facilitates the interpretation of our results.

In summary, the present study confirms that acute PA induces a distinct serum metabolomic phenotype and identifies candidate biomarkers that remain abnormal during the very early post-resuscitation period. These findings extend previous work by applying high-resolution NMR-based serum profiling in a neonatal piglet model and by focusing on the very early post-resuscitation period metabolic state. This can be considered as a significant strength of our study since it provides valuable information regarding the time window early after an asphyctic insult. If validated in human neonates, such metabolite signatures could support early risk stratification and time-sensitive management decisions and contribute to individualized approaches in PA.

## 5. Conclusions

In conclusion, this neonatal piglet study shows that severe asphyxia is associated with a distinct serum metabolic signature and that several abnormalities remain detectable 30 min after resuscitation, indicating incomplete early metabolic recovery. The latter may be useful for biomarker application since the metabolic fingerprint of the insult appears to remain detectable for some time following its presentation, thus allowing for a prolonged time window of diagnosis and assessment and facilitating the diagnosis of cases that were not as straightforward upon delivery. Nevertheless, the lack of reversibility of the metabolomic alterations must be interpreted with caution given the lack of serial measurements over a longer period of time and the inability to define the exact timepoint of the potential recovery of each involved pathway and the subsequent restoration of the respective metabolites’ levels.

Although extensive research has been conducted in the field of metabolomics, it constitutes an inexhaustible source of information for neonatal medicine. In order for certain substances to be used as biomarkers and attain their diagnostic or prognostic value, the initial step would presuppose the measurement and quantification of potentially significant metabolites in controlled circumstances. Future studies incorporating larger cohorts, serial sampling and neurological or histopathological outcome assessment are required to determine whether these early metabolic signatures can improve clinical risk stratification in perinatal asphyxia.

Subsequently, certain metabolomic profiles could be correlated with distinct clinical phenotypes. Elucidating these metabolic signatures could facilitate early, individualized application of the optimal treatment and management that would lead to the most favorable patient outcomes.

## Figures and Tables

**Table 1:** Presentation of the timepoints of blood sampling throughout the experiment based on the animal group.

Animal Category (Number of Animals per Group)	Timepoint of Blood Sampling		
	Baseline	Asphyxia	Final
A (12 piglets)	30 min after initial stabilization		1 h after baseline
B (11 piglets)	30 min after initial stabilization	After hemodynamic compromise	
C (10 piglets)	30 min after initial stabilization	After hemodynamic compromise	Following 30 min of stabilization post-ROSC/Prior to death

A: control, B: asphyxia without resuscitation, C: asphyxia followed by resuscitation. Baseline: 30 min after initial stabilization. Asphyxia: after hemodynamic compromise as defined in the text. Final: approximately 1 h following baseline in Group A animals/after 30 min of stabilization post-ROSC or prior to death if no stabilization post-ROSC in Group C animals. ROSC: return of spontaneous circulation.

**Table 2:** Cumulative doses of anesthetic drugs (propofol, ketamine, cis-atracurium) administered to the animals of the different groups throughout the experimental procedures.

Animal Group	*N*	Propofol (mg/kg)	Fentanyl (μg/kg)	Cis-Atracurium (mg/kg)
**A**	11	30 (mean 29.2 ± 4.8)	20.0 (mean 21.8 ± 4.0)	0.15 (mean 0.25 ± 0.18)
**B**	11	27.8 (mean 29.1 ± 5.7)	20.0 (mean 25.5 ± 8.2)	0.15 (mean 0.22 ± 0.08)
**C**	10	36.0 (mean 36.0 ± 7.6) *	25.0 (mean 26.0 ± 7.0)	0.15 (mean 0.20 ± 0.10)

A: control, B: asphyxia without resuscitation, C: asphyxia followed by resuscitation. *N:* number of animals, values presented: median; in brackets: mean ± SD. * *p* = 0.031 vs. Group B, *p* = 0.024 vs. Group A (Mann–Whitney U).

**Table 3:** AUROC values with bootstrap-derived 95% confidence intervals for key discriminant metabolites, “Asphyxia” vs. “Baseline” comparison (2000 bootstrap iterations).

Metabolite	AUC	95% CI
Lactate	0.943	0.871–1.000
Succinate	0.895	0.779–0.988
Fumarate	0.864	0.733–0.969
Lysine	0.892	0.741–1.000
Hypoxanthine	0.838	0.712–0.940
Isoleucine	0.908	0.819–0.978

AUC: Area Under the Curve, 95% CI: 95% confidence interval.

**Table 4:** AUROC values with bootstrap-derived 95% confidence intervals for key discriminant metabolites, “Baseline” vs. “Resuscitation” comparison (2000 bootstrap iterations, *n* = 10 in Resuscitation group).

Metabolite	AUC	95% CI
Lactate	0.936	0.806–1.000
Lysine	0.895	0.680–1.000
Fumarate	0.893	0.674–1.000
Succinate	0.883	0.698–1.000
Glutamate	0.855	0.728–0.954
Alanine	0.855	0.645–0.985
Hypoxanthine	0.845	0.685–0.970
Choline	0.752	0.520–0.979

AUC: Area Under the Curve, 95% CI: 95% confidence interval.

**Table 5:** Retrospective power analysis for the three primary discriminant metabolites, using Cohen’s *d* computed from observed group means/SDs and achieved power calculated for a two-sample t-test at *α* = 0.05, using the actual group sizes of this study.

Comparison	Metabolite	Cohen’s *d*	Achieved Power (*α* = 0.05)
Asphyxia (*n* = 21) vs. Baseline (*n* = 42)	Lactate	2.93	>0.999
	Succinate	1.95	1.000
	Hypoxanthine	1.44	1.000
Baseline (*n* = 42) vs. Resuscitation (*n* = 10)	Lactate	2.74	1.000
	Succinate	1.82	0.999
	Hypoxanthine	1.79	0.999

Cohen’s *d*: Standardized Mean Difference, *α* (alpha): Significance Level. Baseline: samples obtained 30 min after initial stabilization merged with final samples obtained from Group A animals (control group). Asphyxia: samples obtained after hemodynamic compromise as defined in the text. Resuscitation: samples obtained after 30 min of stabilization post-ROSC or prior to death if no stabilization post-ROSC from Group C animals. ROSC: return of spontaneous circulation.

**Table 6:** Descriptive statistics (median, IQR) and paired Wilcoxon signed-rank test results for key discriminant metabolites, “Asphyxia” vs. “Baseline” comparison. *N* pairs reflects animals with matched samples at both timepoints. FDR-adjusted using Benjamini–Hochberg.

Metabolite	*n* (Asphyxia)	Median (IQR) Asphyxia	*n* (Baseline)	Median (IQR) Baseline	*p* (Paired)	FDR
Lactate	21	861.5 (700.0–1063.6)	42	215.7 (187.8–292.6)	3.815 × 10^−6^	1.144 × 10^−5^
Succinate	21	812.6 (192.7–1212.0)	42	93.1 (84.4–107.5)	5.341 × 10^−5^	5.341 × 10^−5^
Fumarate	21	7.4 (4.5–10.3)	42	1.7 (0.9–2.3)	5.341 × 10^−5^	5.341 × 10^−5^
Lysine	21	80.3 (66.4–91.3)	42	40.6 (34.6–46.8)	2.670 × 10^−5^	5.341 × 10^−5^
Hypoxanthine	21	144.4 (93.3–182.1)	42	71.6 (42.8–82.2)	3.815 × 10^−5^	5.341 × 10^−5^
Isoleucine	21	17.0 (12.0–25.1)	42	35.1 (29.8–42.6)	3.815 × 10^−6^	1.144 × 10^−5^

*n:* number of samples, IQR: Interquartile Range, FDR: False Discovery Rate. Baseline: samples obtained 30 min after initial stabilization merged with final samples obtained from Group A animals (control group). Asphyxia: samples obtained after hemodynamic compromise as defined in the text.

**Table 7:** Descriptive statistics (median, IQR) and paired Wilcoxon signed-rank test results for key discriminant metabolites, “Baseline” vs. “Resuscitation” comparison (*n* = 10 matched pairs).

Metabolite	*n* (Baseline)	Median (IQR) Baseline	*n* (Resuscitation)	Median (IQR) Resuscitation	*p* (Paired)	FDR
Lactate	42	309.0 (243.8–398.0)	10	797.4 (533.6–1166.4)	0.002	0.013
Lysine	42	40.6 (34.6–46.8)	10	81.6 (65.1–102.4)	0.004	0.013
Fumarate	42	1.7 (0.9–2.3)	10	12.6 (6.2–22.5)	0.004	0.013
Hypoxanthine	42	71.6 (42.8–82.2)	10	156.6 (91.0–237.9)	0.014	0.02
Succinate	42	93.1 (84.4–107.5)	10	356.2 (176.8–1119.7)	0.006	0.015
Acetate	42	75.8 (69.2–91.0)	10	119.2 (80.3–168.8)	0.049	0.054
Alanine	42	99.8 (83.3–129.2)	10	163.4 (150.9–184.9)	0.01	0.02
Glutamate	42	198.8 (167.1–235.2)	10	267.1 (248.8–433.1)	0.02	0.024
Choline	42	298.5 (258.9–368.0)	10	553.4 (297.2–630.7)	0.014	0.02

*n:* number of samples, IQR: Interquartile Range, FDR: False Discovery Rate. Baseline: samples obtained 30 min after initial stabilization merged with final samples obtained from Group A animals (control group). Resuscitation: samples obtained after 30 min of stabilization post-ROSC or prior to death if no stabilization post-ROSC from Group C animals. ROSC: return of spontaneous circulation.

**Table 8:** Comprehensive results table, “Asphyxia” vs. “Baseline” comparison. VIP scores from OPLS-DA predictive component (MetaboAnalyst 6.0). Sorted by VIP score.

Metabolite	*n* (Asphyxia)	Median (IQR) Asphyxia	*n* (Baseline)	Median (IQR) Baseline	log_2_FC	*p* (Paired)	FDR	VIP	AUROC	95% CI
Lactate	21	861.5 (700.0–1063.6)	42	215.7 (187.8–292.6)	2.0	3.81 × 10^−6^	1.14 × 10^−5^	1.41	0.943	0.871–1.000
Succinate	21	812.6 (192.7–1212.0)	42	93.1 (84.4–107.5)	3.13	5.34 × 10^−5^	5.34 × 10^−5^	1.36	0.895	0.779–0.988
Lysine	21	80.3 (66.4–91.3)	42	40.6 (34.6–46.8)	0.98	2.67 × 10^−5^	5.34 × 10^−5^	1.31	0.892	0.741–1.000
Fumarate	21	7.4 (4.5–10.3)	42	1.7 (0.9–2.3)	2.16	5.34 × 10^−5^	5.34 × 10^−5^	1.24	0.864	0.733–0.969
Isoleucine	21	17.0 (12.0–25.1)	42	35.1 (29.8–42.6)	−1.05	3.81 × 10^−6^	1.14 × 10^−5^	1.2	0.908	0.819–0.978
Hypoxanthine	21	144.4 (93.3–182.1)	42	71.6 (42.8–82.2)	1.01	3.81 × 10^−5^	5.34 × 10^−5^	1.09	0.838	0.712–0.940

*n:* number of samples, IQR: Interquartile Range, log_2_FC: logarithm base 2 of fold change, FDR: False Discovery Rate, VIP: Variable Importance in Projection, AUROC: Area Under the Receiver Operating Characteristic curve, CI: confidence interval. Baseline: samples obtained 30 min after initial stabilization merged with final samples obtained from Group A animals (control group). Asphyxia: samples obtained after hemodynamic compromise as defined in the text. OPLS-DA: Orthogonal Partial Least Squares Discriminant Analysis.

**Table 9:** Comprehensive results table, “Baseline” vs. “Resuscitation” comparison. VIP scores from OPLS-DA predictive component (MetaboAnalyst 6.0). Sorted by VIP score. * Acetate did not meet the AUROC > 0.75 retention threshold (AUROC = 0.712) and FDR was marginal (*q* = 0.054), included here for completeness given its VIP > 1.0 and reported role in the original discriminant model, but interpreted with appropriate caution.

Metabolite	*n* (Baseline)	Median (IQR) (Baseline)	*n* (Resuscitation)	Median (IQR) Resuscitation	log_2_FC	*p* (Paired)	FDR	VIP	AUROC	95% CI
Lactate	42	309.0 (243.8–398.0)	10	797.4 (533.6–1166.4)	1.37	0.002	0.013	1.52	0.936	0.806–1.000
Lysine	42	40.6 (34.6–46.8)	10	81.6 (65.1–102.4)	1.01	0.004	0.013	1.43	0.895	0.680–1.000
Fumarate	42	1.7 (0.9–2.3)	10	12.6 (6.2–22.5)	2.92	0.004	0.013	1.38	0.893	0.674–1.000
Hypoxanthine	42	71.6 (42.8–82.2)	10	156.6 (91.0–237.9)	1.13	0.014	0.02	1.29	0.845	0.685–0.970
Succinate	42	93.1 (84.4–107.5)	10	356.2 (176.8–1119.7)	1.94	0.006	0.015	1.27	0.883	0.698–1.000
* Acetate	42	75.8 (69.2–91.0)	10	119.2 (80.3–168.8)	0.65	0.049	0.054	1.1	0.712	0.454–0.928
Alanine	42	99.8 (83.3–129.2)	10	163.4 (150.9–184.9)	0.71	0.01	0.02	1.03	0.855	0.645–0.985
Glutamate	42	198.8 (167.1–235.2)	10	267.1 (248.8–433.1)	0.43	0.02	0.024	1.02	0.855	0.728–0.954
Choline	42	298.5 (258.9–368.0)	10	553.4 (297.2–630.7)	0.89	0.014	0.02	1.01	0.752	0.520–0.979

*n*: number of samples, IQR: Interquartile Range, log_2_FC: logarithm base 2 of fold change, FDR: False Discovery Rate, VIP: Variable Importance in Projection, AUROC: Area Under the Receiver Operating Characteristic curve, CI: confidence interval. Baseline: samples obtained 30 min after initial stabilization merged with final samples obtained from Group A animals (control group). Resuscitation: samples obtained after 30 min of stabilization post-ROSC or prior to death if no stabilization post-ROSC from Group C animals. OPLS-DA: Orthogonal Partial Least Squares Discriminant Analysis.

## Data Availability

The raw data supporting the conclusions of this article are available by the corresponding authors on request.

## Abbreviations

The following abbreviations are used in this manuscript:

AMP	Adenosine Monophosphate
ATP	Adenosine Triphosphate
AQ	Acquisition Time
AU	Arbitrary Units
AUROC	Area Under the Receiver Operating Characteristic curve
BBB	Blood–Brain Barrier
BBI	Broad Band Inverse
BCAA	Branched chained amino acids
CI	Confidence Interval
CluPA	Hierarchical Cluster-based Peak Alignment
CNS	Central Nervous System
CPMG	Carr-Purcell-Meiboom-Gill
CSF	Cerebrospinal Fluid
D2O	Deuterium oxide
D/W	Dextrose in Water
ECG	Electrocardiogram
ETT	Endotracheal Tube
FDR	False Discovery Rate
FID	Free Induction Decay
GABA	Gamma-Aminobutyric Acid
GC—MS	Gas Chromatography–Mass Spectrometry
HIE	Hypoxic–Ischemic Encephalopathy
HR	Heart Rate
HSQC	Heteronuclear Single Quantum Coherence
HSQC—DEPT	Heteronuclear Single Quantum Coherence—Distortionless Enhancement by Polarization Transfer
ILCOR	International Liaison Committee on Resuscitation
IQR	Interquartile Range
LC—MS	Liquid Chromatography-Mass Spectrometry
LC-TOFMS	Liquid Chromatography-Time of Flight Mass Spectrometry
Log2FC	log2 Fold Change
MAP	Mean Arterial Pressure
MLEV	Malcolm Levitt’s sequence
MOD	Multiple Organ Dysfunction
MS	Mass Spectrometry
NADH	Nicotinamide Adenine Dinucleotide
NLS	Neonatal Life Support
NMR	Nuclear Magnetic Resonance
NS	Number of Scans
OPLS-DA	Orthogonal Partial Least Squares Discriminant Analysis
PA	Perinatal Asphyxia
PCA	Principal Component Analysis
PQN	Probabilistic Quotient Normalization
ROC	Receiver Operating Characteristic
ROS	Reactive Oxygen Species
ROSC	Return of Spontaneous Circulation
S/N ratio	Signal-to-Noise ratio
SpO2	Peripheral Oxygen Saturation
SW	Spectral Width
TCA cycle	Tricarboxylic Acid cycle
TH	Therapeutic Hypothermia
TOCSY	Total Correlation Spectroscopy
TPPI	Time-Proportional Phase Incrementation with Pulsed Field Gradients
TSP	Trimethylsilylpropanoic acid
UPLC-MS	Ultra-Performance Liquid Chromatography-Mass Spectrometry
UPLC-MS/MS	Ultra Performance Liquid Chromatography coupled to tandem Mass Spectrometry
VIP	Variable Importance in Projection
VLDL	Very Low-Density Lipoproteins
WHO	World Health Organization
XOR	Xanthine Oxidoreductase
